# Microbe-driven elemental cycling enables microbial adaptation to deep-sea ferromanganese nodule sediment fields

**DOI:** 10.1186/s40168-023-01601-2

**Published:** 2023-07-25

**Authors:** Dechao Zhang, Xudong Li, Yuehong Wu, Xuewei Xu, Yanxia Liu, Benze Shi, Yujie Peng, Dadong Dai, Zhongli Sha, Jinshui Zheng

**Affiliations:** 1 Qingdao Key Laboratory of Marine Biodiversity and Conservation, Institute of Oceanology, Chinese Academy of Sciences, Qingdao, 266071 China; 2 Laboratory for Marine Geology, Laoshan Laboratory, Qingdao, 266237 China; 3 University of Chinese Academy of Sciences, Beijing, 100049 China; 4 National Key Laboratory of Agricultural Microbiology, Huazhong Agricultural University, Wuhan, 430070 China; 5 Hubei Key Laboratory of Agricultural Bioinformatics, College of Informatics, Huazhong Agricultural University, Wuhan, 430070 China; 6 Key Laboratory of Marine Ecosystem Dynamics, Ministry of Natural Resources & Second Institute of Oceanography, Ministry of Natural Resources, 310012 Hangzhou, China; 7 Key Laboratory of Marine Geology and Environment, Institute of Oceanology, Chinese Academy of Sciences, Qingdao, 266071 China

## Abstract

**Background:**

Ferromanganese nodule-bearing deep-sea sediments cover vast areas of the ocean floor, representing a distinctive habitat in the abyss. These sediments harbor unique conditions characterized by high iron concentration and low degradable nutrient levels, which pose challenges to the survival and growth of most microorganisms. While the microbial diversity in ferromanganese nodule-associated sediments has been surveyed several times, little is known about the functional capacities of the communities adapted to these unique habitats.

**Results:**

Seven sediment samples collected adjacent to ferromanganese nodules from the Clarion–Clipperton Fracture Zone (CCFZ) in the eastern Pacific Ocean were subjected to metagenomic analysis. As a result, 179 high-quality metagenome-assembled genomes (MAGs) were reconstructed and assigned to 21 bacterial phyla and 1 archaeal phylum, with 88.8% of the MAGs remaining unclassified at the species level. The main mechanisms of resistance to heavy metals for microorganisms in sediments included oxidation (Mn), reduction (Cr and Hg), efflux (Pb), synergy of reduction and efflux (As), and synergy of oxidation and efflux (Cu). Iron, which had the highest content among all metallic elements, may occur mainly as Fe(III) that potentially functioned as an electron acceptor. We found that microorganisms with a diverse array of CAZymes did not exhibit higher community abundance. Instead, microorganisms mainly obtained energy from oxidation of metal (e.g., Mn(II)) and sulfur compounds using oxygen or nitrate as an electron acceptor. Chemolithoautotrophic organisms (*Thaumarchaeota* and *Nitrospirota* phyla) were found to be potential manganese oxidizers. The functional profile analysis of the dominant microorganisms further indicated that utilization of inorganic nutrients by redox reactions (rather than organic nutrient metabolism) is a major adaptive strategy used by microorganisms to support their survival in the ferromanganese nodule sediments.

**Conclusions:**

This study provides a comprehensive metagenomic analysis of microbes inhabiting metal-rich ferromanganese nodule sediments. Our results reveal extensive redundancy across taxa for pathways of metal resistance and transformation, the highly diverse mechanisms used by microbes to obtain nutrition, and their participation in various element cycles in these unique environments.

Video Abstract

**Supplementary Information:**

The online version contains supplementary material available at 10.1186/s40168-023-01601-2.

## Introduction

Sediments at depths greater than 500 m (deep-sea sediments) cover approximately two thirds of the earth’s surface, and represent one of its largest ecosystems. They are characterized by a huge diversity of habits including soft-sediment abyssal plains, seamounts, continental slopes, and submarine canyons [1, 2]. As these habitats are highly heterogeneous, the interactions between biosphere and geosphere are likely to differ among the various sites. After decades of exploration, much knowledge has accumulated about the diversity and function of life in these habits. However, knowledge of the relationships between the adaptations of life to these special environments and the biogeochemical cycling of organic and inorganic elements remains limited [3–5].

The deep-sea nodule province, characterized by the presence of large quantities of ferromanganese nodules, is one of the best known special habitats in the abyss [6]. The ferromanganese nodules have an average size of 1–5 cm, and mainly comprise iron and manganese oxides, although other metals including nickel, cobalt, copper, titanium, and rare earth elements are present at low levels [6, 7]. With the exception of the Mediterranean and the Red Sea, ferromanganese nodules are formed on or just below the vast, sediment-covered abyssal plains of the global ocean, at depths ranging from 100 to 6000 m [8]. However, they mainly aggregate in the Clarion–Clipperton Fracture Zone (CCFZ; in the North Pacific Ocean) where the total dry mass of mature and large nodules is conservatively estimated to be 21 billion tons [6, 9]. In the past few decades, research has shown that deep-sea ferromanganese nodules are not solely created through non-biogenic mechanisms (such as hydrogenetic and diagenetic processes), but are also influenced by biologically mediated processes involving microorganisms [10, 11]. However, how microbes survive in these special habitats and contribute to the metallic element transformation is unclear.

Mining of ferromanganese nodules has been largely driven by commercial and strategic interests in critical metals for high-tech industries [6, 12]. The Clarion–Clipperton Zone’s abyssal seafloor is the world’s largest and most economically promising area for deep-sea mining of polymetallic nodules [10]. Mining, however, has a significant impact on the natural state of the ecosystem by altering the physical and chemical properties of the environment [13, 14]. Regardless of the mining method used, subsequent restoration of the sediment surface may take many years or centuries [13]. Consequently, before mining is permitted in the CCFZ, it is necessary to understand the natural microbial diversity and its role in elemental cycling within the sediments.

Previous studies using cultivation-dependent and cultivation-independent approaches have revealed the microbial community composition and diversity in sediments from the Pacific Nodule Province [15–21]. However, as these insights are primarily inferred from 16S rDNA analysis, the metabolic potential and capacities of these sediment microorganisms remain unclear, particularly the uncultured component. In this study, we performed deep metagenomic sequencing of seven sediment-associated communities from surface Fe–Mn sediments from the CCFZ in the East Pacific Ocean. We successfully reconstructed 179 high-quality metagenome-assembled genomes (MAGs) belonging to 21 bacterial phyla and 1 archaeal phylum. These MAGs enabled understanding of the metabolic potential of all major taxa around Fe–Mn nodules in these deep-sea sediments.

## Results and discussion

### Sediment sampling and major elemental compositions

Sediment samples were collected in August 2018 from seven sites (depth: 4983–5211 m) in the CCFZ polymetallic nodule province in the eastern equatorial Pacific Ocean during cruise DY 135-E2, as part of China’s 50th Ocean Expedition (Additional file 1: Fig. S1). The sediment samples contained Fe, Mn, Zn, Cu, Cr, Pb, As, and very low levels of Cd and Hg (Additional file 2: Table S1). The sediments were rich in Fe (Fe_56_ and Fe_57_) and Mn, which were the two major elements. The Fe content was highest among all the metallic elements, and exceeded the Mn content by approximately 12-fold. This was consistent with a previous report that the Fe content exceeds that of Mn (Fe/Mn ratio≈6) in the South Pacific Gyre [22]. The content of these metals among the seven samples was very similar, suggesting that the environment of the sampling region was stable. To investigate the adaptation of microorganisms to the environment with high metal concentrations, we employed metagenomic approaches to explore the metabolic potential of microorganisms.

### Microbial community composition

Metagenomic sequencing of DNA data obtained from the seven sediment samples generated 874.19 Gbp of clean data, which were assembled into 49.46 Gbp of contiguous segments (contigs). The total length of contigs ≥ 1 kb was 18.34 Gbp (Additional file 2: Table S2). We used the gene encoding ribosomal protein S3 (RPS3) in contigs ≥ 1 kb to explore the microbial community diversity, as described previously [23]. In total, 4298 *rpS3* gene sequences were retrieved from the seven metagenomic assemblies, and were grouped into 2267 nonredundant clusters that approximated species groups (SGs). A total of 22 phylum-level lineages were detected, and the community composition among sample sites showed high similarity (Additional file 2: Table S3; Additional file 1: Fig. S2). Given the high level of similarity in the community composition among the seven sample sites where the content of different metals was stable, the results of our study may provide a reference for future study of microbial function and diversity in other deep-sea polymetallic nodule provinces.

The most relatively abundant bacterial lineages were *Proteobacteria* (classes *Alphaproteobacteria* and *Gammaproteobacteria*) and *Planctomycetes*, followed by *Acidobacteria*, *Chloroflexi*, *Gemmatimonadetes*, and *Nitrospirae* while *Thaumarchaeota* was the most abundant archaeal lineage across all the sampling sites. These results are consist with several microbial community studies of similar habitats based on 16S rRNA sequences [14, 21, 24–26].

### Recovery of MAGs and dominant microorganisms in ferromanganese nodule sediments

**Fig. 1:**
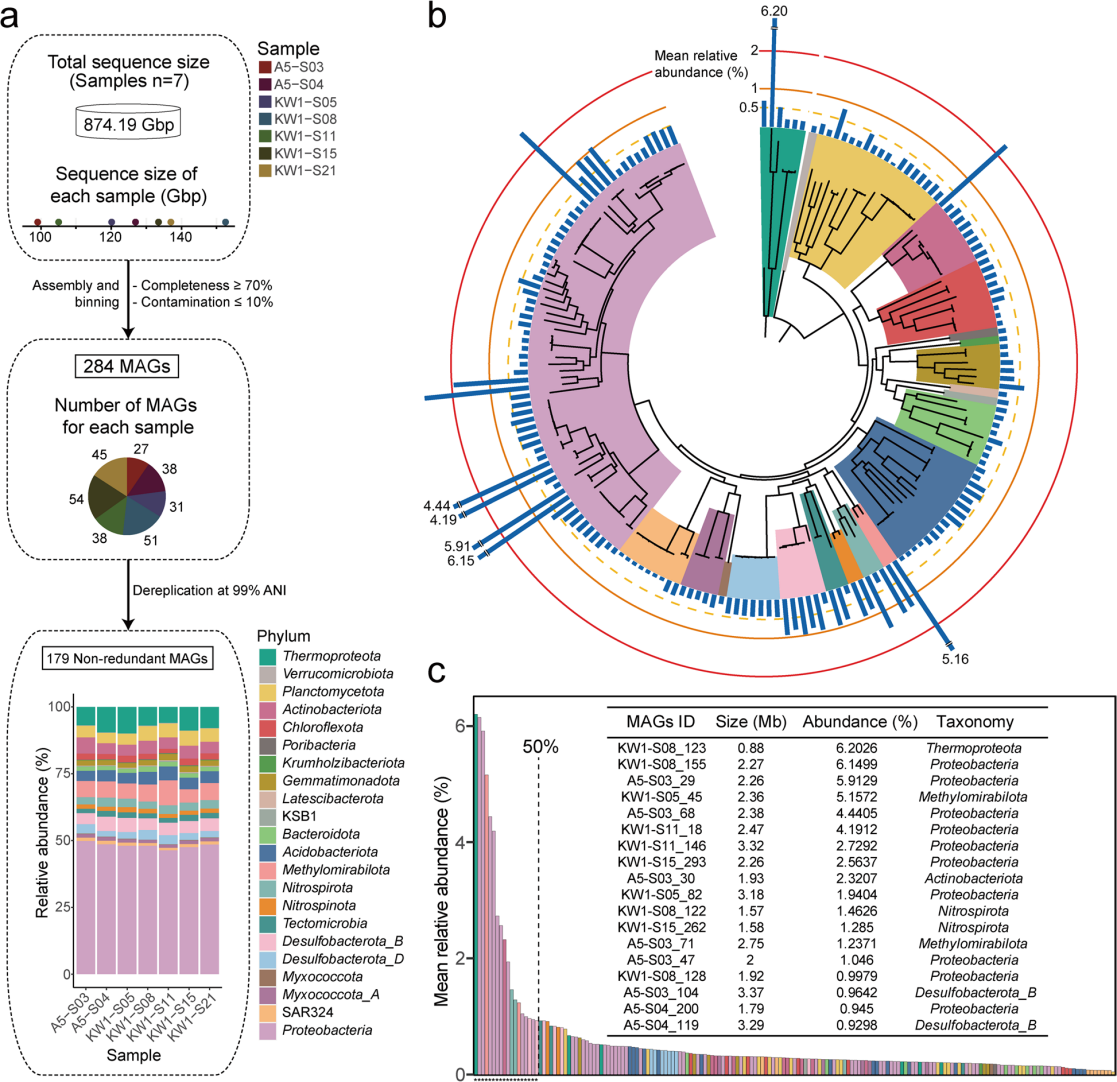
Maximum likelihood tree for 179 high-quality nonredundant MAGs and dominant microbial components in deep-sea ferromanganese nodule sediment fields. **a** Overview of metagenomics sequencing, binning, and diversity. Based on 874.19 Gbp of data, assembly, binning, and filtering were implemented to recover a total of 284 MAGs, and following dereplication 179 nonredundant MAGs were retained. The stacked bar chart shows the relative abundance of these MAGs in seven samples. Colors correspond to phyla. **b** Combined archaeal and bacterial phylogenetic tree. The archaeal tree was constructed based on a concatenated alignment of 122 single-copy marker proteins, which was inferred using GTDB-Tk. And 120 single-copy marker proteins were used for the bacterial tree. The concentric bar plot represents the mean relative abundance. **c** MAGs ranked by mean relative abundance across all samples. The sum of the relative abundance of dominant MAGs accounted for at least 50% of the total abundance; these MAGs are marked with asterisks on the *x*-axis. Details of dominant MAGs are shown in the inserted table

To infer the functional roles of the microbes in these unique niches, we applied genome-centric methods to the deep-sequenced metagenomic data. A total of 284 MAGs were recovered by assembly and binning (≥ 70% completeness and ≤ 10% contamination), with 27–54 MAGs obtained per sediment sample (Fig. 1a). After dereplication at 99% average nucleotide identity (ANI), 179 representative high-quality nonredundant MAGs were selected. The predicted genome sizes ranged from 0.71 to 6.39 Mbp, with estimated completeness of 70.23–98.48% and contamination of 0–9.89% (Additional file 2: Table S4). These 179 MAGs were taxonomically assigned to 22 phyla, and 88.8% of the genomes were unclassified at the species level, indicating the existence of previously unexplored microbial taxa (Fig. 1b). Among the 179 MAGs, 64 (36%) were assigned to *Proteobacteria*. Other phyla, represented by more than 10 MAGs, included *Planctomycetota* (18/179; 10%) and *Acidobacteriota* (16/179; 9%). The six archaeal MAGs were classified to the phylum *Thermoproteota* (same as *Thaumarchaeota* in NCBI taxonomy; Additional file 1: Fig. S3), many of which carry out chemolithoautotrophy by oxidizing ammonia in the deep sea, and provide primary carbon for the food chain [22]. We found that the composition and abundance of MAGs were very similar among samples (Additional file 2: Table S5), which is consistent with the results based on *rpS3* gene sequences. To define dominant microorganisms in the ferromanganese nodule sediments, we examined the 18 high relative abundance MAGs that constituted the top 50% of all 179 reconstructed genomes (Fig. 1c). These belonged to the phyla *Thermoproteota*, *Proteobacteria*, *Methylomirabilota*, *Actinobacteriota*, *Nitrospirota*, and *Desulfobacterota_B*. The most abundant MAG (KW1-S08_123; 6.2%) with smallest genome size among dominant microorganisms belonged to the family *Nitrosopumilaceae* (*Thermoproteota*).

### Metal resistance and cycling

Besides maintaining homeostasis with respect to metals including iron, copper, manganese, and zinc (which play a fundamental role in numerous intracellular processes such as enzyme catalysis, protein folding, and signaling), microorganisms in deep-sea sediments of the polymetallic nodule province have to develop effective ways to avoid toxicity associated with the presence of various metals [27]. To maintain the balance of metal ion concentrations, microbes can use metal transporters to transport metals into various cellular membrane compartments [28]. Efflux mechanisms are critical in the resistance of bacteria to a diverse array of heavy metals [29]. Among these mechanisms, exporting P-type ATPases can efficiently promote the detoxification of heavy metal cations by exporting inorganic substrates from the cytoplasm to the external environment or periplasm [30]. In addition, some microorganisms have evolved energy metabolism that is reliant on using either reducing or oxidizing metals as electron donors or acceptors, respectively [31]. This unique ability can play vital roles in the biogeochemical cycles of metals.

#### Mn

**Fig. 2:**
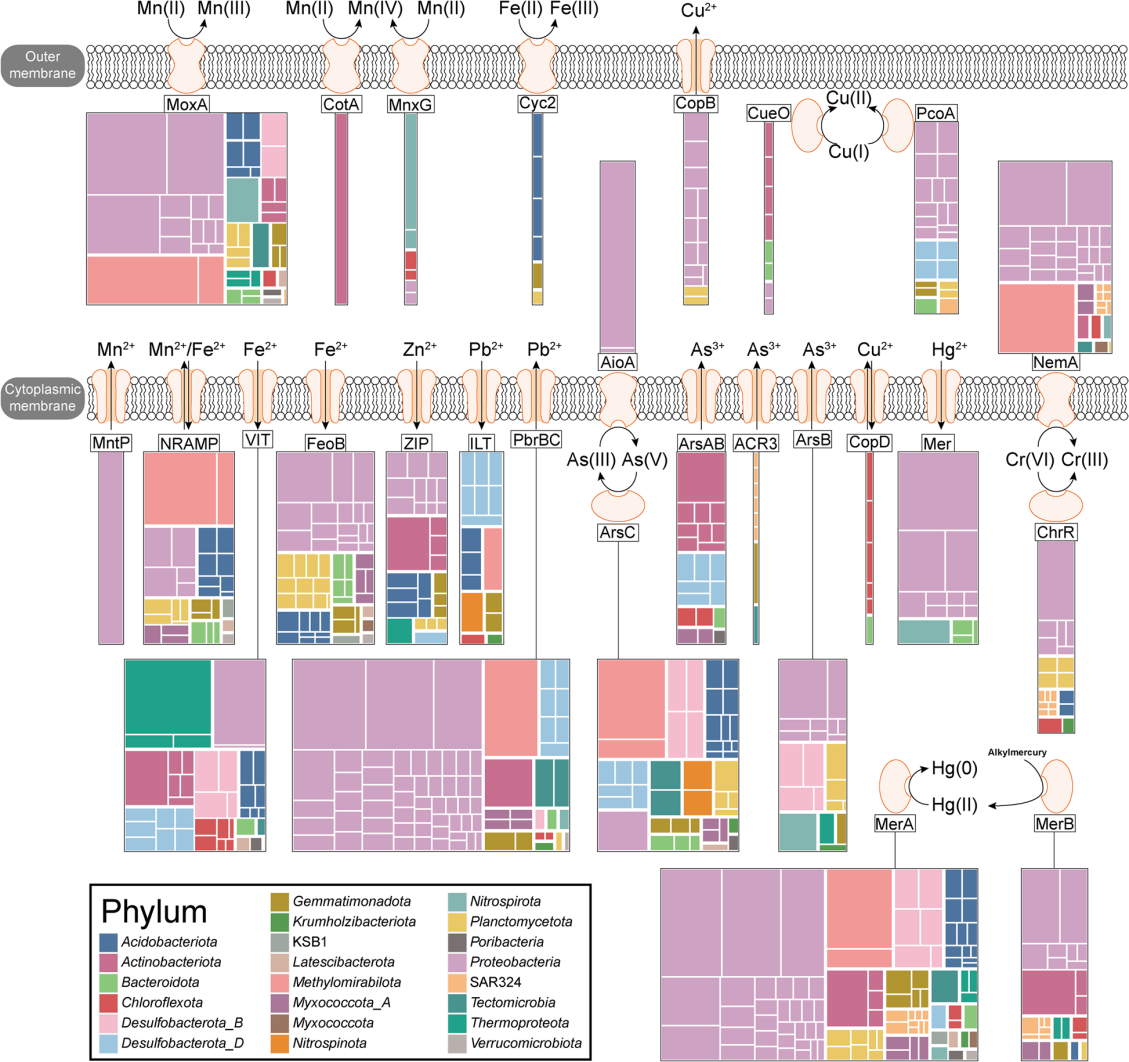
Metal transport and redox reactions of MAGs. The yellow colors shown on the bacterial membrane, periplasm, or cytoplasm represent proteins, with the corresponding names written in the adjacent black boxes. The treemaps indicate the composition of MAGs which contain the genes encoding the related proteins, with a block representing each MAG. The size corresponds to the mean relative abundance, and the color corresponds to classification at the phylum level. Some proteins are not shown due to low abundance or incomplete function, including AioB, Cut, CymA, MtrC, and Sulfocyanin. Detailed information can be found in Additional file 2: Table S6 and S7

Biochemical characterization has identified the natural resistance-associated macrophage protein (NRAMP) homologs in both Gram-negative and Gram-positive bacteria as transporters of divalent metal ions, with a notable affinity for Mn(II), and a lesser affinity for Fe(II) [32, 33]. MntH, a type of transporter belonging to NRAMP family, serves as a significant Mn^2+^ importer in bacteria [34]. A previous study has experimentally demonstrated *Salmonella typhimurium* and *Escherichia coli* NRAMPs are highly selective Mn(II) transporters [35]. In this study, the gene encoding NRAMP was widely detected in 32 MAGs belonging to *Methylomirabilota*, *Proteobacteria*, *Acidobacteriota*, *Planctomycetota*, *Myxococcota_A, Gemmatimonadota*, *Bacteroidota*, KSB1, *Latescibacterota*, and *Verrucomicrobiota* (Fig. 2 and Additional file 2: Table S6). In addition, we found that the *mntP* gene (formerly *yebN*) encoding a putative manganese efflux pump occurred in one *Proteobacteria* MAG.

Mn(II)-oxidizing microorganisms play a significant role in the biogeochemical cycling of Mn and other elements by catalyzing the oxidation of soluble Mn(II) to Mn(III, IV) oxides. These microbes have been predicted to greatly increase the rate of Mn mineralization by several orders of magnitude relative to abiotic Mn oxidation [36]. Mn(IV) oxides have been found in diverse marine environments such as ferromanganese nodules in the deep sea, ore deposits, metalliferous sediments and hydrothermal mounds near spreading centers, and deep-ocean ferromanganese crusts located on seamounts [37]. Multicopper oxidases (MCOs) are a versatile class of enzymes that employ copper as a cofactor to catalyze the oxidation of a wide range of substrates, including diverse metals like Fe(II) and Mn(II) [38]. So far, bacterial MCOs involved with Mn(II) oxidation have been genetically identified in three bacteria, *Bacillus* sp. strain SG-1 (*mnxG*) [39], *Leptothrix discophora* SS-1(*mofA*) [40] and *Pedomicrobium* sp. strain ACM3067 (*moxA*) [41]. Another MCO (CotA) from a highly active Mn(II)-oxidizing strain *Bacillus pumilus* WH4 was responsible for Mn(II) oxidation based on the direct observation of Mn(II) oxidation with the heterologously expressed protein CotA [42]. Among the MAGs, we found three genes (*mnxG*, *moxA*, and *cotA*) encoding MCO (Additional file 2: Table S7). We detected the *mnxG* gene in six bacterial MAGs, the *moxA* gene in 44 bacterial and three archaeal MAGs, and the *cotA* gene in one bacterial MAG. Among the dominant MAGs in our study, KW1-S08_155, A5-S03_68, and KW1-S11_18 (*Alphaproteobacteria*), KW1-S05_45 and A5-S03_71 (*Methylomirabilota*), and KW1-S08_122 (*Nitrospirota*) contained the *moxA* gene, and A5-S03_30 (*Actinobacteriota*) contained the *cotA* gene. This suggests that these dominant microorganisms were probably involved in Mn oxidation.

A high proportion of the chemolithoautotrophic bacterial phylum *Nitrospirota* (*Nitrospirae* in NCBI taxonomy) has been reported in the Fe–Mn crust and Fe–Mn crust endolithic biofilm samples from the Rio Grande Rise, Southwestern Atlantic Ocean, and is a group considered potentially involved in manganese oxide formation [25]. Interestingly, in our study one dominant *Nitrospirota* MAG (KW1-S08_122) contained *mnxG* and *moxA* genes, and one *Nitrospirota* MAG (KW1-S15_218) harbored the *mnxG* gene. Based on prediction of conserved domains, we found that both MnxG and MoxA proteins from *Nitrospirota* MAGs contained cupredoxin (Additional file 1: Fig. S4). Cupredoxins, referred to as blue copper proteins, play a role in electron transport reactions and contain a type-1 copper at their active site [43].

The potential for manganese oxidation by archaea have been reported in some studies. For example, the dominant clone types grouped with mesophilic archaea in both the *Crenarchaeota* and *Euryarchaeota* were found in ferromanganese cave deposits [44]. Marine Group I (MGI) *Thaumarchaeota*, an archaeal group, have been recognized as one of the most common chemoautotrophs thriving in the deep sea [45]. MGI *Thaumarchaeota* found on a ferromanganese nodule in the ultra-oligotrophic South Pacific Gyre are considered potential candidates for manganese oxidation due to their possession of several genes coding for copper-containing proteins, including MCO, which is utilized by most known manganese oxidizers [22]. We found that three *Thaumarchaeota* MAGs (KW1-S08_208, KW1-S11_157, and KW1-S15_227) contained the *moxA* gene. The overall fold of an extremely thermostable multicopper oxidase from the hyperthermophilic archaeon *Pyrobaculum aerophilum* (McoP) was comprised of three cupredoxin-like domains [46]. In this study, we further found that these three MoxA proteins from *Thaumarchaeota* MAGs contained cupredoxin based on prediction of conserved domains (Additional file 1: Fig. S4). It is important to highlight that while no Mn(II)-oxidizing archaea have been experimentally confirmed to date, our genomic-level analysis results have revealed the potential of microbial communities for future efforts for isolation and cultivation.

A co-culture of two bacterial species including “*Candidatus Manganitrophus noduliformans*” which is affiliated to the phylum *Nitrospirota*, was reported to demonstrate Mn(II) oxidation-dependent chemolithoautotrophic exponential growth [47]. Recently, comparative genomic analyses suggested that the family “*Candidatus Manganitrophaceae*” utilized chemolithotrophic pathways for Mn(II) oxidation [48]. The phyla *Thaumarchaeota* and *Nitrospirota* are known to be chemolithoautotrophic organisms [45, 47]. In this study, five MAGs belonging to these two phyla were found to contain the genes encoding manganese(II) oxidase, among which the KW1-S08_122 MAG was highly abundant (1.46%). Our results show that sediment chemolithoautotrophic microorganisms may also play a significant role in manganese oxidation, in addition to major heterotrophic/mixotrophic manganese-oxidizing microorganisms. Nevertheless, further experimental evidence is needed to confirm whether they can actually use manganese oxidation as an energy source, given that only one chemoautotrophic manganese oxidizer belonging to *Nitrospirota* has been reported [47].

#### Fe

To ensure a supply of essential iron, microorganisms have evolved intricate mechanisms to maintain iron homeostasis, which involves the use of integral membrane proteins that transport ferrous iron [31]. The vacuolar iron transporter (VIT) proteins are involved in transport of iron and have been studied mainly in plants and yeasts, although homologs have also been identified in other eukaryotes and in bacteria and archaea [49]. For example, the membrane-bound ferritin (MbfA) protein belonging to the erythrin-vacuolar iron transport (Er-VIT1) family may function physiologically as an iron efflux transporter [29, 50]. In this study, 40 MAGs possessed the gene encoding VIT. The prokaryotic membrane protein FeoB is essential for Fe(II) uptake in bacteria [51]. The *feoB* gene was identified in 53 MAGs and was widespread in the *Proteobacteria*, *Planctomycetota*, *Acidobacteriota*, and *Bacteroidota*.

In order to prevent the mineralization of the cytoplasm within the cell, iron oxidation takes place at the outer surface of the cell [31]. The cyc2 gene, which encodes a putative iron oxidase, is a reliable indicator of iron oxidation ability and is found in various Fe-oxidizing lineages including acidophiles and neutrophils [52]. The Cyc2-based iron oxidation pathway in neutrophilic Fe-oxidizing bacteria (for example, the well-studied neutrophilic chemolithotrophs *Gallionellaceae* and *Zetaproteobacteria*) is widespread and plays a significance role in marine environments where iron mineralization occurs [52]. The most extensively researched group of iron-oxidizing bacteria is the acidophilic *Acidithiobacillus ferrooxidans*. In this microorganism, electrons generated from the oxidation of Fe(II) to Fe(III) are gathered by iron oxidase Cyc2, and then transferred to a periplasmic rusticyanin [53]. Of six MAGs containing the *cyc2* gene, four belonged to *Acidobacteriota*, and two belonged to *Planctomycetota* and *Gemmatimonadota*. Sulfocyanin, a blue copper-haem protein, facilitates electron transfer during iron oxidation in *Ferroplasma* strains [54]. In addition, it has been proposed that a predicted sulfocyanin in *Sulfobacillus thermosulfidooxidans* strain ST may also serve as a component of the electron transport chain involved in iron oxidation [55]. In this study, two *Gemmatimonadota* MAGs (A5-S04_112 and KW1-S21_123) contained the gene encoding the sulfocyanin which may have an important role mediating Fe(II) oxidation.

Microbial dissimilatory iron reduction (DIR) is a crucial process in suboxic environments including subsurface soils and sediments where it mediates iron cycling. A novel flavin-based electron transport chain in diverse Gram-positive bacteria has been reported to support growth using an extracellular electron acceptor (ferric iron) [56]. Iron was at very high concentrations in the studied sediment samples, and we detected genes encoding the proteins involved in this novel extracellular electron transfer (EET) process. The genes encoding various proteins involved in EET were abundant, but we did not detect any MAG that contained the genes encoding all proteins for the complete EET (Ndh2, DmkB, DmkA, EetB, EetA, FmnA, FmnB, PplA) [56]. There are two categories of NADH dehydrogenase present in bacteria: the NADH-1 enzyme complex and NADH-2 which is a single-subunit enzyme encoded by the *ndh* gene [57]. The *ndh2* gene was found in 74 MAGs and was widespread in the *Proteobacteria*, *Thermoproteota*, *Methylomirabilota*, *Desulfobacterota_B*, *Desulfobacterota_D*, *Planctomycetota*, and *Actinobacteriota.* During the EET process, electrons are transferred by NADH-2 from NAD to demethylmenaquinone (DMK), which is synthesized by proteins DmkA and DmkB, and finally to FMN groups on PplA or free flavin shuttles [56]. We found that 20% (35/179) of MAGs contained the gene encoding DmkA, and 78% (139/179) possessed the gene encoding DmkB. Based on these results, we speculate that the genes encoding a unique extracellular electron transfer using ferric iron as an electron acceptor may be contained in the studied MAGs (Additional file 1: Fig. S5). We speculate that the absence of genes encoding all the proteins of a complete EET in the MAGs is because of differences in the EET for these microbes, or presence of proteins that perform alternative functions. For example, although we did not detect the *pplA* gene, 78 MAGs harbored the *fmnB* gene (Additional file 1: Fig. S5c), suggesting that FmnB was very abundant. In a model of the molecular basis of EET, it was reported that FmnB uses FAD secreted by RibU and FmnA to post-translational modification of PplA [56]. We speculate that an unknown protein may replace PplA and perform the transfer of electrons in the MAGs studied. In addition, it is noteworthy that we found that one *Planctomycetota* MAG (KW1-S08_227) contained the *mtrC* gene encoding an outer membrane c-type cytochrome, and the *Proteobacteria* MAG (A5-S03_68) contained the *cymA* gene encoding an inner membrane periplasmic tetraheme quinol dehydrogenase, both of which play important roles in Fe(III) reduction process [58, 59]. These results indicate that many types of electron transfer occur in the studied environment and that Fe(III) acts as the terminal electron acceptor in a bacterial respiratory electron transport chain under suboxic conditions.

Our results also indicate that Fe(II)-oxidizing and Fe(III)-reducing bacteria in the sediments associated with polymetallic nodules may be metabolically flexible. It is worth noting that among those MAGs that harbored genes responsible for oxidation of Fe(II), five also contained the *moxA* gene responsible for oxidation of Mn(II), including MAG (A5-S03_93), MAG (KW1-S11_180), and MAG (KW1-S15_197) (*cyc2* and *moxA*); and MAG (KW1-S21_123) and MAG (A5-S04_112) (*sulfocyanin* and *moxA*). We speculate that these microorganisms may have two independent oxidizing factors and can oxidize both Mn(II) and Fe(II).

Overall, we found that MAGs contained more genes responsible for iron reduction than iron oxidation (524 iron reduction genes and 9 iron oxidation genes, Additional file 2: Table S8), and iron may occur mainly in the ferric state as an electron acceptor in sediments, rather than ferrous iron. With respect to iron transport, microbes mainly uptake Fe(II) rather than excreting it, given that VIT (40/179 MAGs) and FeoB (53/179 MAGs) have the potential to uptake Fe(II). These results suggested that the concentration of Fe(II) may be low, and iron may occur mainly as Fe(III) in sediment samples.

#### Cu/Zn

Copper is a key micronutrient for bacteria, serving as an indispensable cofactor for redox cuproenzymes. However, copper becomes toxic at high concentrations, and microorganisms have developed some intrinsic mechanisms to resist excess copper, such as pumping copper ions out of the cytoplasm and oxidizing Cu^+^ to Cu^2+^ in the periplasmic space [60]. Fourteen MAGs (12 *Proteobacteria* and two *Planctomycetota*) contained the *copB* gene encoding P-type Cu^2+^ transporter, and six MAGs (five *Chloroflexota* and one *Bacteroidota*) contained the *copD* gene encoding a copper resistance protein (Additional file 2: Table S6). In addition, the *cut* gene encoding the copper homeostasis protein was identified in KW1-S08_89 (*Proteobacteria*). We found the *cueO* gene encoding the multicopper oxidase in eight MAGs; it was most common among the *Actinobacteriota, Bacteroidota*, and *Proteobacteria*. In addition, 24 MAGs contained the *pcoA* gene encoding a periplasmic multicopper oxidase; this catalyzes the oxidation of Cu^+^ to less toxic Cu^2+^ as part of the copper resistance response. Twelve MAGs were found to contain both the *copB* and *pcoA* genes, indicating that these microorganisms use a coordinated approach of oxidation and efflux to maintain copper homeostasis. This suggests that these two genes work synergistically to regulate copper levels within the cell. It has been reported that the rate of Mn(II) oxidation catalyzed by MCO is increased by the addition of Cu(II) [22], so it is possible that those manganese-oxidizing microorganisms oxidizing Mn(II) via MCO use Cu(II) present in the sediments.

Zinc is an essential element as a cofactor for numerous enzymes. Zinc import and export are two critical processes that maintain zinc homeostasis in bacteria besides intracellular zinc binding and zinc-sensing [61]. Members of Zn^2+^-Fe^2+^ permease (ZIP) family were involved in Zn(II) import [61] and 28 MAGs harbored the gene encoding ZIP in this study.

#### As/Pb/Cr/Hg

Arsenic is a toxic metal commonly distributed in the environment, which requires microorganisms to have effective resistance mechanisms and redox enzymes [62]. The arsenite oxidase (AioAB) oxidizes the more toxic arsenite to the less toxic arsenate in the periplasm [63]. The *aioA* and *aioB* genes were found in two *Proteobacteria* MAGs (Additional file 2: Table S7). One is MAG (KW1-S21_27) which contained *aioA* and *aioB* genes and the other is MAG (KW1-S08_155) which contained *aioA* gene. Forty-five MAGs harbored *arsC* gene encoding cytoplasmic arsenate reductase responsible for arsenate reduction from As(V) to As(III). Many MAGs harbored transmembrane carrier pump-related genes encoding arsenite export protein ACR3, As(OH)_3_/H^+^ antiporter ArsB, and arsenite extrusion pump ArsAB (Additional file 2: Table S6) by which As(III) can be pumped out of the cell. It is worthy to note that fourteen MAGs simultaneously contained both protein ArsC and As(III) export pump, suggesting that these bacteria may resist Arsenic by synergy of reduction and efflux.

Lead detoxification, achieved through active efflux and sequestration, represents a well-characterized mechanism for mitigating the toxic effects of lead [64]. Hynninen et al. proposed a mechanism in which the P_1B_-type Pb(II) efflux ATPase PbrA is responsible for transporting Pb(II) outside the cytoplasm, while PbrB produces inorganic phosphate to sequester it [64]. In this study, 16 MAGs possessed the *ilt* gene encoding the iron/lead transporter [65], and 77 MAGs (most affiliated with *Proteobacteria*) contained the *pbrBC* gene encoding the lead resistance fusion protein.

Microbial resistance to the highly toxic Cr(VI) can be achieved through reduction to Cr(III), a less toxic form, and this is a common and effective microbial detoxification mechanism [66]. Chromate reductases (e.g., ChrR and NemA) catalyzing the reduction of Cr(VI) to Cr(III) can be found in the cytoplasm or membrane of bacterial cells [66]. The *chrR* gene was identified in 21 MAGs belonging to *Proteobacteria*, *Planctomycetota*, SAR324, *Acidobacteriota, Chloroflexota*, and *Krumholzibacteriota*, and the *nemA* gene was detected in 39 MAGs, mostly *Proteobacteria*, suggesting that these bacteria may mainly carry out the reduction of Cr(VI) to Cr(III).

In the bacterial mercurial compounds detoxification system, organomercurial lyase (MerB) and mercuric reductase (MerA) can sequentially demethylate methylmercury (MeHg) to Hg(II) and reduce Hg(II) to Hg0, respectively [67]. Fourteen MAGs contained *mer* gene encoding the mercuric ion pore superfamily, which catalyze Hg(II) uptake from the periplasm, and its transport across the membrane into the cytoplasm for reduction by MerA [68]. In addition, we found that 26 MAGs contained the *merB* gene. Furthermore, the gene *merA* was found in 96 MAGs, suggesting that most microbial communities have the capacity to reduce Hg(II) to gaseous Hg(0).

Our results show that microorganisms in ferromanganese nodule sediments have developed many intrinsic mechanisms to resist heavy metals, for example, oxidation (Mn), reduction (Cr and Hg), efflux (Pb), synergy of reduction and efflux (As), synergy of oxidation and efflux (Cu). The transformation of Mn, Fe, Cu, As, Cr, and Hg through redox reactions of heterotrophic and chemolithoautotrophic microorganisms in the sediments may simultaneously affect the cycles of other elements including carbon, nitrogen, and sulfur, depending on environmental fluctuations of electron donors and acceptors.

### Carbon metabolism

**Fig. 3:**
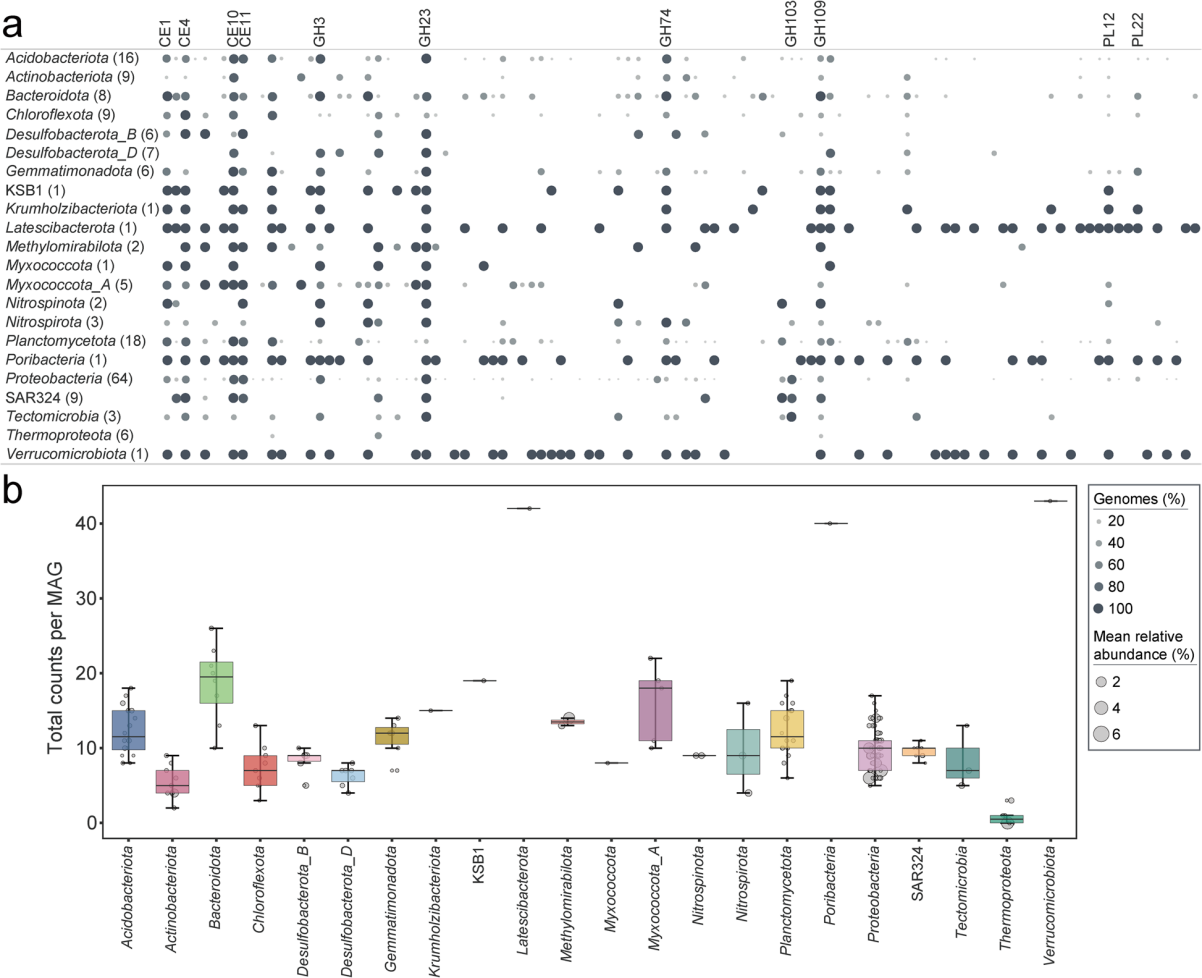
Carbohydrate-active enzymes (CAZymes) detected in MAGs. **a** Number of MAGs which contained the genes encoding CAZymes. The bubble plot summarizes percentage of genomes within each phylum which contained the genes encoding carbohydrate esterases (CEs), glycoside hydrolases (GHs), and polysaccharide lyases (PLs). Numbers in parentheses indicate the total number of genomes for each phylum. Detailed data can be found in Additional file 2: Table S10. **b** Counts of CAZymes of MAGs per phylum. The box plots display the minimum value, first quartile (Q1), median, third quartile (Q3), and maximum value for the dataset for each phylum. Points represent the total number of CAZymes detected in each MAG, and the point sizes correspond to the mean relative abundance

In order to analyze the potential of microorganisms to degrade carbohydrates in sediments, genes encoding carbohydrate-active enzymes (CAZymes) were identified. Our analysis showed that among the CAZymes within the sediment metagenome, GH (glycoside hydrolase) was the most abundant class present, followed by CE (carbohydrate esterase) and PL (polysaccharide lyase) (Fig. 3). With respect to enzymes of GH family, there was marked enrichment of GH23 (143 MAGs), GH3 (106 MAGs), GH109 (86 MAGs), GH103 (69 MAGs), and GH74 (66 MAGs) (Additional file 2: Table S9-10). It has been reported that there was a high incidence of GH23 (lytic transglycosylase) and GH74 (xyloglucanase) in the metagenome from dynamic Guaymas Basin hydrothermal sediments [69]. In total, we identified 654 putative CE genes, particularly CE10 (149 MAGs), CE11 (123 MAGs), CE4 (112 MAGs), and CE1 (95 MAGs). While CE10 and CE1 include esterases acting on non-carbohydrate substrates, CE4 includes chitin and peptidoglycan deacetylases. Among the PL families, PL22 (oligogalacturonate lyase/oligogalacturonide lyase; 29 MAGs) and PL12 (heparin-sulfate lyase; 27 MAGs) dominated. Chitin, a polysaccharide second only to cellulose in abundance in nature, is characterized by its high insolubility, yet chitinases demonstrate an exceptional ability to directly hydrolyze this polymer into lower molecular weight chitooligomers [70]. As the enzymes predicted to be involved in degrading chitin (GH23 and CE4) were very enriched, we speculate that chitin may be an important carbon nutrient in the ultra-oligotrophic deep-sea environment.

*Poribacteria*-related genomes and members of the candidate phylum *Latescibacterota* have been detected in culture-independent surveys in marine habitats including marine sediments, pelagic water column, and deep-sea hydrothermal vent plumes [71, 72]. The bacterial phylum *Verrucomicrobia* is found in almost all marine environments and plays an important role in global carbon cycling and degrading various polysaccharides [73, 74]. We found that *Poribacteria* (KW1-S08_28), *Latescibacterota* (KW1-S15_112), and *Verrucomicrobiota* (KW1-S08_24) with highest diversity of CAZyme types had very low abundance (0.18%, 0.20%, 0.15%, respectively). This result showed that microorganisms with a wide variety of CAZymes were not dominant in the ferromanganese nodule sediments.

In addition to elucidating the degradation of complex carbon substrates by microorganisms in sediments, we further examined the enzymes employed by these microorganisms for the utilization of small carbon compounds, including carbon monoxide (CO), methanol, formaldehyde, lactate, acetate, and propionate (Additional file 1: Fig. S6; Additional file 2: Table S11). Acetyl-CoA synthetase (Acs) can facilitate the conversion of acetate into acetyl-CoA, which is an essential molecule involved in various metabolic pathways [75, 76]. Within our study, we identified the *acs* gene in 162 MAGs that belong to 22 phyla. This finding suggests that the microorganisms present in the sediments possess potential to utilize acetate as a carbon source, despite the fact that Acs has multiple substrate specificities [77]. CO oxidation is facilitated by aerobic carbon monoxide dehydrogenase (CoxSML), a heterotrimeric enzyme that employs CoxL as its catalytic subunit [78]. The gene *coxL* was detected in 100 MAGs, indicating CO probably provides a significant source of energy in the studied environment. However, a recent study has suggested that CoxL may metabolize other substrates [23], emphasizing the need for further research to explore the microbial utilization of CO.

### Nitrogen cycling

Nitrogen interconversions from nitrate (NO_3_^−^) to ammonia/ammonium (NH_3_/NH_4_^+^) creates the nitrogen cycle which plays a critical role in establishing the geochemical environment in deep-sea sediments [79]. To infer the capacity for nitrogen cycling in polymetallic nodule sediments, we analyzed MAGs for metabolic genes involved in this process.

**Fig. 4:**
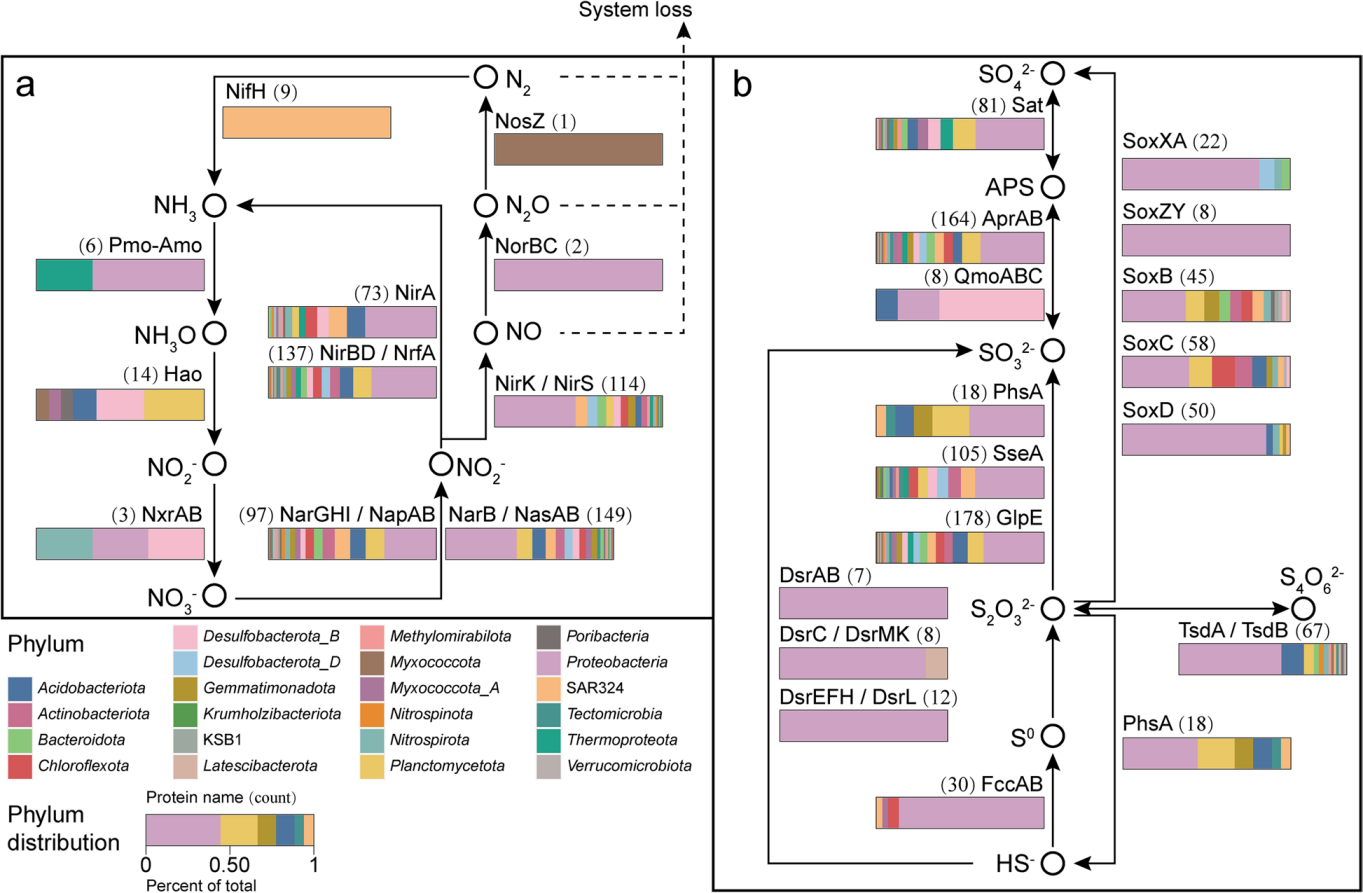
Nitrogen and sulfur metabolic transformations among MAGs. Composition of MAGs from sediment metagenomes having the ability to process nitrogen- and sulfur- containing compound (shown in a and b, respectively). Horizontal stacked histograms indicate proportions of genomes for each function. Numbers in parentheses represent the total number of genomes performing each function

In the environment, NO_3_^−^ levels are influenced by the microbially driven processes of nitrate reduction which can be either an assimilatory nitrate reduction or dissimilatory nitrate reduction process. In the dissimilatory denitrification process, the reduction of NO_2_^−^ to NO is catalyzed by two nitrite reductases (NirK and NirS) [80]. Approximately 64% of MAGs from 18 phyla contained the *nirK/nirS* genes, which indicates the potential for microbial reduction of NO_2_^−^ to NO in this system (Fig. 4; Additional file 2: Table S12). Two organisms within the *Gammaproteobacteria* (MAGs KW1-S15_293 and KW1-S05_58) possessed the *norBC* gene encoding nitric oxide reductase, which convert NO to N_2_O. Only one MAG (KW1-S05_58) within the *Gammaproteobacteria* harbored all genes responsible for the reduction of NO_3_^−^ to N_2_O. Reduction of N_2_O is the final step in the denitrification pathway and represents the release of the nitrogen compound back into the atmosphere. We only identified the *nosZ* gene encoding nitrous oxide reductase (functional for yielding N_2_) in the KW1-S15_267 MAG within the *Myxococcota* phylum. These results suggest that denitrifying bacteria are phylogenetically diverse in the sediments and may be responsible for the release of NO. Furthermore, we found that 41% (73/179) of MAGs possessed *narGHI*/*napAB* and *nirBD*/*nrfA* genes responsible for dissimilatory nitrate reduction, and 35% (63/179) of MAGs contained *narB*/*nasAB* and *nirA* genes responsible for assimilatory nitrate reduction. Genomes in polymetallic nodule sediments were found to be enriched with genes required for both assimilatory nitrate reduction and dissimilatory nitrate reduction processes, suggesting that microbial communities had a potential to use nitrate as electron acceptor.

Ammonia-oxidizing microorganisms, which include both ammonia-oxidizing bacteria (AOB) and ammonia-oxidizing archaea (AOA), catalyze the first step of nitrification by transforming NH_3_ to NO_2_^−^ through hydroxylamine, using the enzymes ammonia monooxygenase and hydroxylamine dehydrogenase [81]. Previous studies have reported that dissolved NH_3_ in seawater is an important nutrient in Fe–Mn crusts, and is likely to be one of the energy sources for sustaining the surface sediment ecosystem [25]. Four reconstructed *Gammaproteobacteria* MAGs, including KW1-S21_28 in the family *Nitrosomonadaceae* (order *Burkholderiales*; order *Nitrosomonadales* in NCBI taxonomy), were predicted to have *pmo-amo* gene encoding the enzyme ammonia monooxygenase. Bacterial and archaeal *amoA* genes have also been detected in the ferromanganese crusts and sediment samples of old seamounts from the northwestern Pacific [26]. We found that two MAGs (KW1-S08_121 and KW1-S08_123) belonging to the family *Nitrosopumilaceae* (*Thermoproteota*), contained *pmo-amo* gene (which is consistent with reports that *Nitrosopumilaceae* MAGs are likely to be putative aerobic ammonium-oxidizing archaea [82, 83]). Fourteen MAGs within the *Planctomycetota*, *Desulfobacterota_B*, *Acidobacteriota*, *Poribacteria, Myxococcota*, and *Myxococcota_A* contained the *hao* gene encoding hydroxylamine dehydrogenase, which is required for the oxidation of hydroxylamine to NO_2_^−^. The second step of nitrification, the oxidation of NO_2_^−^ to NO_3_^−^, is executed by nitrite-oxidizing bacteria using nitrite oxidoreductase (NxrAB). The *nxrAB* gene was found within the genomes of KW1-S11_176 (*Alphaproteobacteria*), KW1-S15_262 (*Nitrospiraceae*), and A5-S04_119 (*Desulfobacterota_B*; formerly known as the class *Deltaproteobacteria*).

The SAR324 group of *Deltaproteobacteria* is ubiquitous in the deep sea and has previously been detected as a potential chemolithoautotroph on the surface of a ferromanganese nodule from the ultra-oligotrophic South Pacific Gyre [22]. In this study, we found that nine MAGs belonging to the SAR324 group contained the *nifH* gene encoding nitrogenase iron protein, indicating SAR324 may participate in N_2_ fixation, which is a source of nitrogen input to the sediments.

Our results show that microbial communities in the sediments possess diverse metabolic genes involved in nitrogen cycling, including assimilatory nitrate reduction, dissimilatory nitrate reduction, nitrification, and nitrogen fixation. As 64% of MAGs contained the *nirK/nirS* genes encoding nitrite reductase and only 1% of MAGs possessed the *norBC* gene encoding nitric oxide reductase in denitrification metabolism, we speculate that the sediments in our study are likely to be a significant source of NO to the seawater. Nitrate may be an important electron acceptor in addition to oxygen and Fe(III) in this environment. Considering the low number of MAGs involved in ammoxidation and nitrite oxidation, we speculate that the primary source of nitrate is seawater rather than nitrogen metabolism by microorganisms in the sediment.

### Sulfur metabolism

Sulfur cycling is a major biogeochemical process in marine sediments. Sulfur compounds are important oxidants or reductants in microbial respiration, and mainly catalyzed by sulfate-reducing bacteria (SRB) and sulfur-oxidizing bacteria (SOB) in sediments [84]. In addition, sulfur disproportionation is a chemolithotrophic microbial process in which elemental sulfur, thiosulfate, and sulfite are oxidized or reduced, producing hydrogen sulfide and sulfate [85].

To assess whether the sediment organisms participated in the dissimilatory sulfite reductase (dsr) pathway or reverse dissimilatory sulfite reductase (rdsr) pathway, we looked for the genes encoding enzymes involved in these pathways, including sulfate adenylyltransferase (Sat), adenylylsulfate reductase (AprAB), dissimilatory sulfite reductase subunit α/β (DsrAB), and quinone-interacting membrane-bound oxidoreductase subunits A, B, and C (QmoABC) [86]. All these genes were detected in the sediment metagenomes, suggesting that these bacteria may play a role in sulfate/sulfite reduction or the sulfur oxidation pathway. To further confirm their roles in the sulfur cycle, we searched the genomes for the *dsrD* gene, which is absent in bacteria that use the rdsr pathway for sulfur oxidation [86, 87]. We did not detect the *dsrD* gene in the context of *dsrAB*, *dsrC*, *dsrL*, *dsrEFH*, and *dsrMK*, which may indicate the potential presence of the rdsr pathway for sulfur oxidation in this system (Fig. 4; Additional file 2: Table S12). In addition, the *dsrL* and *dsrEFH* genes, which are commonly present in sulfur oxidizers but absent in SRB [88, 89], were found in four *Gammaproteobacteria* MAGs (KW1-S15_234, KW1-S05_77, W1-S05_58, and A5-S04_105) and twelve *Gammaproteobacteria* MAGs, respectively. Given the absence of *dsrD* and occurrence of the *dsrL* and *dsrEFH* genes, we speculate that these microorganisms in our samples may be capable of oxidizing sulfide to sulfate via the rdsr pathway for sulfur oxidation. We further searched the MAGs for the *fccAB* gene, which encodes sulfide dehydrogenase comprising a large sulfide-binding flavoprotein (FccB) and a small cytochrome c (FccA) [90]. The gene *fccAB* was found in 30 MAGs including those of *Proteobacteria* (26/30), *Chloroflexota*, SAR324, and *Myxococcota_A*, suggesting these microorganisms in the sediments may metabolize sulfur and form elemental sulfur and/or polysulfides.

Thiosulfate (S_2_O_3_^2−^) is an intermediate sulfur compound and constitutes a key junction in the network of many sulfur metabolic pathways [91]. The metabolic genes (*soxXA*, *soxB*, *soxC*, *soxD*, and *soxZY*) involved in thiosulfate oxidation via the SOX pathway were found in this study; in particular, 45 MAGs affiliated to 13 phyla possessed the *soxB* gene which is the most widespread marker among known marine SOB [84]. Interestingly, we detected two MAGs (KW1-S21_27 and KW1-S08_46) that harbored all the genes responsible for the SOX pathway, indicating their potential for carrying out the entire thiosulfate oxidation process. These organisms harboring sox genes are presumed to mediate oxidative parts of the sulfur cycle in the sediments. The bifunctional enzyme thiosulfate dehydrogenase/tetrathionate reductase (TsdA) can bidirectionally convert tetrathionate to thiosulfate [92]. Meanwhile, the diheme cytochrome c TsdB, while not reactive with thiosulfate itself, acts as the electron acceptor for TsdA [92, 93]. Sixty seven MAGs classified as representing 13 phyla (61% *Proteobacteria*) contained *tsdA* or *tsdB* genes. The Tetrathionate intermediate (S4I) pathway as one of thiosulfate oxidation pathways consists of two enzymes, thiosulfate: quinol oxidoreductase ( DoxDA) and tetrathionate hydrolase (TetH) [94]. We did not detect the *doxDA* gene and the *tetH* gene in the MAGs, suggesting that the S4I pathway was not prevalent among the microorganisms in our samples.

Bacterial sulfur disproportionation occupies a central position in the oxidative sulfur cycle and is a chemolithotrophic process that involves the conversion of sulfur compounds in intermediate valence states into sulfate and sulfide as final products [95]. The *sseA* and *glpE* genes encoding 3-mercaptopyruvate sulfurtransferase and thiosulfate sulfurtransferase which are responsible for thiosulfate disproportionation to sulfite [96], were detected in 105 and 178 MAGs, respectively. It is worth noting that all MAGs containing the *sseA* gene also contain the *glpE* gene, which indicates the metabolic flexibility of these MAGs in relation to sulfur disproportionation. Interestingly, we detected three archaeal MAGs (KW1-S15_144, KW1-S08_121, KW1-S08_123; in the order *Nitrososphaerales*) containing *sseA* and *glpE* genes, suggesting that archaea may participate in thiosulfate disproportionation. In addition, we found that 18 MAGs within the *Proteobacteria*, *Planctomycetota*, *Gemmatimonadota*, *Acidobacteriota*, *Tectomicrobia*, and SAR324 contained the *phsA* gene encoding the putative thiosulfate reductase which is required in the production of sulfite or sulfide from thiosulfate [97].

Thus, metagenomic analyses revealed a complex rdsr pathway for sulfur oxidation network in sediments. Among dominant microorganisms, we found that five *Proteobacteria* MAGs (A5-S03_29, KW1-S11_146, KW1-S05_82, KW1-S08_128, and A5-S04_200) contained numerous genes involved in the oxidation of sulfide, thiosulfate, and sulfite, suggesting the potential for energy to be obtained by oxidation. The rdsr pathway for sulfur oxidation was likely to be coupled to nitrate reduction through microorganisms using nitrate as an electron acceptor, with sulfide, thiosulfate, and sulfite being electron donors.

**Fig. 5:**
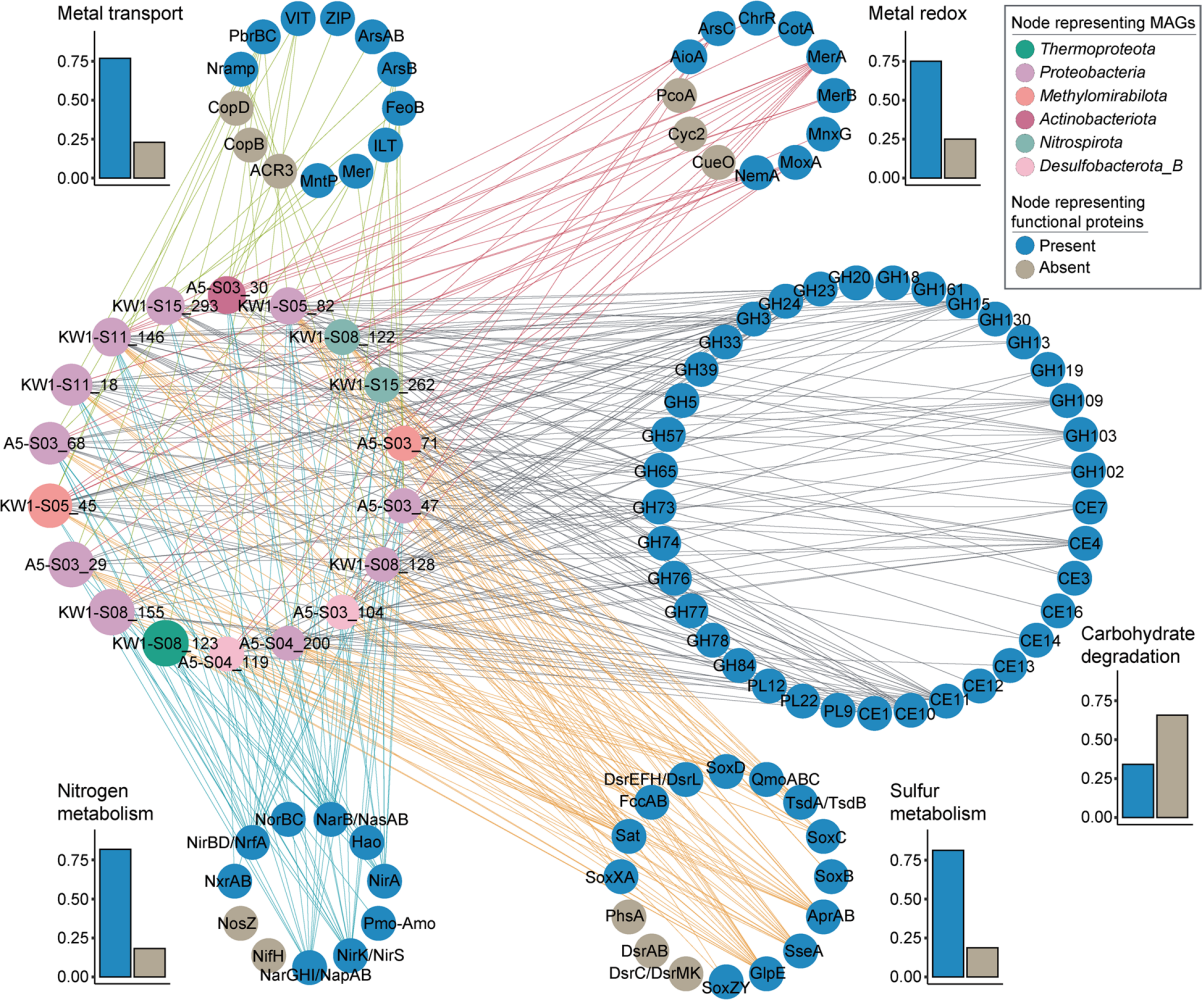
Overview of the metabolic functions of dominant microbial components in deep-sea ferromanganese nodule sediment fields. In the network, nodes in the left middle circle indicate dominant MAGs, the colors represent phylum-level classification, and the circle sizes correspond to abundances. Other nodes represent predicted functions including metal transport and redox potential, carbohydrate degradation, and nitrogen and sulfur metabolism. Connected blue nodes indicate that the genes were present in at least one dominant MAG, and the gray nodes indicate the absence of the genes in all dominant MAGs (gray nodes for carbohydrate degradation are not shown). The histograms show the proportion of detected and undetected genes for each functional category in dominant MAGs

We assessed the 18 high relative abundance MAGs for the presence of functional genes for metal, nitrogen, sulfur, and carbohydrate metabolism. Subsequently, the proportion of gene types present in these dominant MAGs was calculated relative to the total number of gene types within each functional category (Fig. 5). This analysis was performed to showcase the genetic diversity exhibited by these 18 MAGs across various metabolic pathways. We found that the proportion of the genes types involved in metal, nitrogen, and sulfur metabolism was very high (ranging from 75 to 82%), while the occurrence of CAZymes was relatively low (34%). We randomly selected 18 MAGs in 100 replicates among the remaining MAGs and calculated the proportions within different functional categories. Interestingly, the result showed that the remaining MAGs also have similar characteristics to the dominant MAGs, such as a high proportion of genes involved in metal, nitrogen, and sulfur metabolism, and a lower proportion of CAZymes (Additional file 1: Fig. S7). It is worth noting that the proportion of CAZymes contained in the dominant MAGs is much lower than the average of the proportions of CAZymes contained in the remaining MAGs. These results suggested these dominant microorganisms contained relatively few genes involved in the degradation and assimilation of carbohydrate substrates in ultra-oligotrophic deep-sea environments, energy for growth is obtained mainly through redox reactions involving metals, nitrogen, and sulfur. We speculate that inorganic nutrients oxidation may constitute a very important way for many of these microorganisms to obtain energy.

## Conclusions

**Fig. 6:**
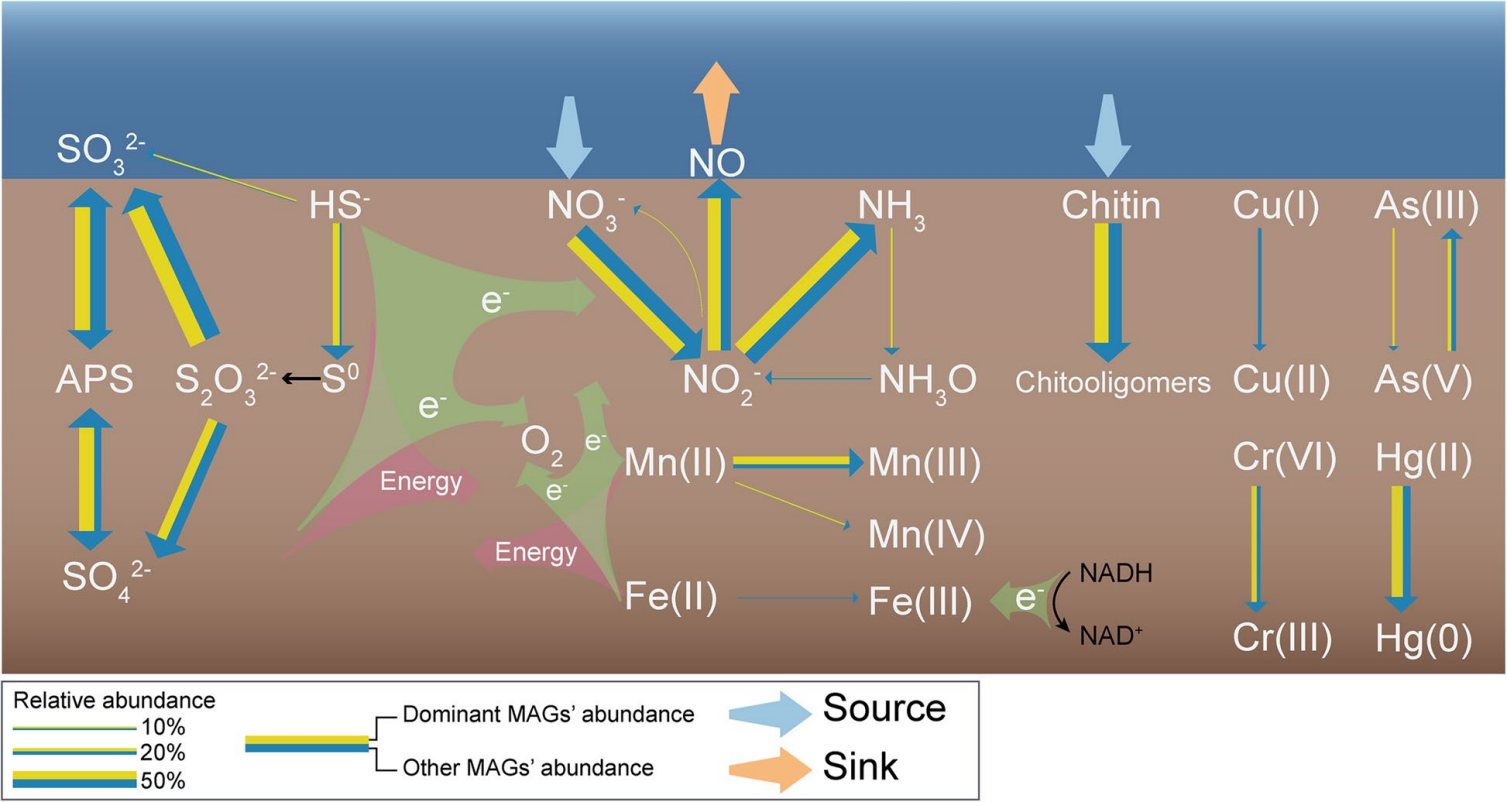
Proposed microbial-dominated ecological functions in deep-sea ferromanganese nodule sediment fields. The width of lines represents the total relative abundance of dominant and other MAGs performing each function. The box model was applied to the ecosystem, simplifying it to a reservoir for chemical materials. The materials marked as sources entered the reservoir, and the materials marked as sinks left the reservoir. In the figure, the oxidation that releases electrons may represent a way to obtain energy from chemoautotrophy

Our research has provided the first comprehensive study of the metabolic capacities of microorganisms in ferromanganese nodule sediments. Investigation of core metabolic genes in MAGs from these sediments provides the first evidence for the roles of these distinct microbes in metal, nitrogen, and sulfur cycling. As part of the study, we proposed a conceptual model for the ecology of the microorganisms in the sediments (Fig. 6). Specifically, our results suggest that within these metal-rich sediment environments, heterotrophic and chemolithoautotrophic microorganisms had developed mechanisms of resistance to heavy metals including metal efflux (Mn, Cu, As, and Pb), adsorption uptake (Fe, Cu, Zn, Pb, and Hg), and metal biotransformation by enzymatic redox (Mn, Fe, Cu, As, Cr, and Hg). For Mn(II) resistance, the Mn(II) was mainly oxidized to Mn(III) or Mn(IV) oxide by manganese-oxidizing microorganisms and little was transported, which highlights the essential nature of the oxidation reaction in sustaining life in this energy-limited system. The discovery of genes for oxidoreductases including Mn(II) oxidase, Fe(III) reductases, Cr(IV) reductases, As(III) oxidase, and Hg(II) reductases provide important genetic resources having potential applications in bioremediation of heavy metals. From our analysis of the MAGs involved in the nitrogen cycling, we can infer that nitrate was the primary electron acceptor and reduced predominantly to NO that was discharged into the seawater. We found that numerous bacteria are involved in a complex rdsr pathway for sulfur oxidation network in sediments. Interestingly, we found that chemolithoautotrophic microorganisms may obtain energy from Mn(II) oxidation, nitrification, thiosulfate disproportionation, and oxidation of sulfide and sulfite. Analysis of the dominant microorganisms showed that the MAGs comprising the top 50% in terms of relative abundance carried more functional genes for metal, nitrogen, and sulfur metabolism, and had fewer CAZymes. This further indicates microbial potential to mainly utilize inorganic nutrients in sediments rather than organic nutrients, and highlights the adaptability of microorganisms in ultra-oligotrophic deep-sea environmental conditions.

## Materials and methods

### Sediment sampling and element analysis

Sediment samples were collected from seven sites in the CCFZ polymetallic nodule province in the eastern equatorial Pacific Ocean. On board the research vessel, the surface sediments (0–5 cm) were collected using a box corer and transferred to sample collection bottles and immediately stored at − 80 °C until analyzed. The sediment samples were digested using a mixture of HNO_3_, HF, and H_2_O_2_ in a high-pressure microwave digestion system at 190 °C for 30 min, and the elemental contents were determined by inductively coupled plasma mass spectrometry (ICP-MS, Thermo iCAP Q) [98]. The accuracy and precision of analytical method were evaluated by analyzing the certified reference material GBW07429 [98].

### DNA extraction, sequence preprocessing, and metagenomic assembly

For each sample, DNA was extracted from 10 g of sediment using the DNeasy PowerMax Soil Kit (Qiagen, Germany). The quality and quantity of genomic DNA were checked using gel electrophoresis and a Qubit 2.0 fluorometer (Invitrogen, Carlsbad, CA, USA). DNA sequencing was performed using an Illumina NovaSeq platform (2 × 150 bp). The quality of raw reads was globally assessed using FastQC software [99]. Trimmomatic v0.38 [100] was used to trim low-quality sequences and the first 10 bp of reads. After trimming, 874.19 Gbp of total reads was retained (943.28 Gbp of raw reads). The high-quality read sets for seven samples were individually assembled using MEGAHIT v1.2.2-beta [101] with default parameters. Assembly quality was evaluated using QUAST [102]. A total of seven assemblies were filtered to exclude contigs smaller than 1 kb using pullseq v1.0.2 (https://github.com/bcthomas/pullseq). On filtered contigs, open reading frames (ORFs) were called using Prodigal v2.6.3 [103] with the following parameters: -p meta.

### rpS3 gene identification, clustering, and classification

The *rpS3* gene sequences were identified by searching all predicted proteins using HMMER3 [104] with a HMM score threshold of 40, and the HMM for the alignment of 2249 *rpS3* gene marker sequences were obtained from the reference [23]. Across all samples, a total of 4298 *rpS3* gene sequences were recognized. We clustered these sequences using USEARCH [105] with the following parameters: -cluster_fast all_rpS3.fasta -sort length -id 0.99 -maxrejects 0 -maxaccepts 0 -centroids rpS3_centroids.fasta -clusters cluster_dir/rpS3_. Each RPS3 protein cluster approximated a species group (SG).

The relative abundance of each SG was calculated using the following method. First, we compared the length of contigs encoding proteins in each protein cluster to extract the longest contig, which served as a mapping target for calculating abundance. Reads from each sample were then mapped to the longest contig of each SG using BWA v0.7.17-r1188 [106] for mapping, samtools [107] for sorting, and picard [108] for removing duplicates. Mapped reads were filtered using the CoverM v0.6.1 “filter” with the following parameters: –min-read-percent-identity 99 –min-read-aligned-percent 75. The coverage of each contig was calculated using the “mean” method in CoverM v0.6.1 “contig”. The relative abundance of the contig representing each SG in each sample was equal to its coverage divided by the total coverage of all contigs.

Taxonomic classification of *rpS3* SGs at the phylum level was determined based on the phylogenetic relationship between our sequences and *rpS3* gene reference sequences [23]. Briefly, we used MAFFT [109] to align our 2267 *rpS3* gene sequences representing all SGs to the 2249 *rpS3* gene reference sequences. Columns containing more than 95% gap positions in the alignment were removed using trimAl [110]. A maximum likelihood tree was inferred using IQ-TREE [111] with the following parameters: -m TEST -alrt 1000 -bb 1000. According to the position of our *rpS3* gene sequences on the reference phylogenetic tree, their classification was recognized manually.

### Genome binning and quality control

The individual metagenome assemblies (contigs ≥ 2.5 kb) were binned into MAGs using MetaBAT v2.12.1 [112], on the basis of tetranucleotide frequencies and differential coverage profiles. Coverage profiles were summarized from sequences map files, which were generated by mapping all reads from each sample to the corresponding assemblies using BWA v0.7.17-r1188 [106], samtools [107], and picard [108]. The resulting MAGs were refined using RefineM [113] in the following two steps to reduce contamination. First, contaminating contigs for each MAG were identified based on genomic properties, and removed. Second, removal of contaminating contigs was based on taxonomic assignments using the GTDB database release R95 [114] as a reference database.

### MAG dereplication and taxonomic classification

All 1697 refined MAGs were dereplicated using the “dereplicate” command in dRep [115], using the following parameters: -comp 70 -con 10. For our dataset, 179 high-quality nonredundant MAGs were obtained. Taxonomic assignment of these MAGs was inferred using GTDB-Tk v1.3.0 [116], based on the GTDB database release R95 [114] and using “Classify workflow” with default parameters. To ensure accuracy, the GTDB-based classification was checked using *rpS3* gene-based classification at the phylum level for each MAG containing *rpS3* gene sequences. Assembly quality for each genome was evaluated using QUAST [102].

### Calculation of relative abundance

To calculate the relative abundance of each high-quality nonredundant MAG in each sample, we used BWA v0.7.17-r1188 [106] for mapping, samtools [107] for sorting, and picard [108] for removing duplicates, to map reads from each individual metagenome to the 179 MAGs. The resulting BAM files were filtered using the CoverM v0.6.1 (https://github.com/wwood/CoverM) “filter” to remove low-quality mappings (identity < 95%; aligned length < 75%). The relative abundance of each genome in each sample was then calculated using CoverM v0.6.1 “genome” with “relative_abundance” method.

### MAG annotation

Protein-coding genes of the 179 MAGs were predicted using prodigal v2.6.3 [103], and rRNAs of the MAGs were identified using barrnap v0.9 [117] with the following parameters: –kingdom arc/bac (based on classification of MAGs) –reject 0.3 –evalue 1e − 05. tRNAs of the MAGs were identified using tRNAscan-SE v2.0.7 [118] with -A/-B option.

The membrane metal transport proteins encoded in MAGs were identified using BLASTP [119] (e-value threshold: 1e − 20) against the Transporter Classification Database (TCDB) [120]. FeGenie [121] was used to annotate proteins for iron redox reactions. Proteins for other metal redox reactions were identified using BLASTP [119] (e-value threshold: 1e − 20) against the custom databases. For the accuracy of annotations, proteins involved in metal transport and redox reactions were checked using BLASTP [119] against NCBI’s nonredundant (NR) database [122] (Additional file 2:Table S13). Additionally, proteins for nitrogen and sulfur cycling were identified using BLASTP [119] (e-value threshold: 1e − 20) against NCycDB [123] and SCycDB [124], respectively. We also checked the annotations involved in nitrogen and sulfur metabolism based on the domains of these proteins predicted by eggNOG-mapper v2.1.2 [125] (Additional file 2:Table S14). CAZymes encoded in MAGs were identified using HMMER3 [104] against the dbCAN v8 HMM database [126] with the following parameters: -E 1e-14 –domE 1e − 14. The enzymes responsible for the metabolism of small carbon compounds in MAGs are derived from the KEGG Ortholog (KO) [127] annotations, which are predicted using the eggNOG-mapper v2.1.2 [125]. Conserved domains of MnxG and MoxA proteins were detected using NCBI’s CD-search tool [128].

### Visualization

Figures in the manuscript were generated using custom R scripts [129], ggplot2 [130], Adobe Illustrator (http://www.adobe.com/au/products/illustrator.html), cytoscape [131], tableau (https://www.tableau.com/zh-cn), and IBS [132].

## Supplementary Information

**Additional file 1:** **Fig. S1**. Location of sampling sites in the CCFZ polymetallic nodule province in the eastern equatorial Pacific Ocean. **Fig. S2.** rpS3 species group classification and abundance. a, Relative abundance of all species groups (SGs) in each phylum. Red represent the most abundant SGs, whose combined average relative abundance was at least 25% of that of all SGs. ‘Other’ comprises phyla accounting for ≤ 5 SGs. ‘Unclassify’ indicates that some SGs could not be classified by phylogenetic methods. b, Relative abundance of members of the microbial community at the phylum level across seven samples. **Fig. S3.** Comparison of GTDB- and rpS3 gene-based classifications of the MAGs. The left side of the Sankey diagram represents the GTDB classification at the phylum level for MAGs containing *rpS3* gene sequences. The right side represents *rpS3* gene-based classification using NCBI taxonomy. **Fig. S4.** Schematic diagram of conserved domains of MnxG and MoxA proteins. Blue regions represent cupredoxin superfamily, and purple region represents LysM superfamily. **Fig. S5.** Flavin-based extracellular electron transfer (EET) in MAGs. a, EET model with iron as an electron acceptor. The process of electron transfer was drawn in accordance with [58]. In brief, the electron transfer path from NAD to Fe(III) includes Ndh2, DMK, EetB, EetA, and FMN groups on PplA, or free flavin shuttles. b, Synthesis of DMK via the proteins DmkB and DmkA. c, Post-translational modification of PplA. FAD is secreted via FmnA and RibU, and FmnB uses FAD to post-translationally modify PplA. The treemaps indicate the composition of MAGs which contained the genes encoding the proteins. DHNA, 1,4-dihydroxy-2-naphthoyl-CoA; DMK, demethylmenaquinone; FAD, flavin adenine dinucleotide; FMN, Flavin mononucleotide; IPP, isopentenyl pyrophosphate. **Fig. S6.** Functional roles of MAGs in the metabolism of small carbon compounds. The horizontal stacked histograms illustrate the distribution of genomes encoding each function. The numbers within parentheses correspond to the total number of genomes that exhibit each function. MaxF, calcium-dependent methanol dehydrogenase subunit 1 (K14028); FdhA, glutathione-independent formaldehyde dehydrogenase (K00148); Ldh, L-lactate dehydrogenase (K00016); LdhA, D-lactate dehydrogenase (K03778); Acs, acetyl-CoA synthetase (K01895); Pct, propionate CoA-transferase (K01026); CoxL, aerobic carbon monoxide dehydrogenase large subunit (K03520). **Fig. S7.** Functional profile of the remaining MAGs other than the dominant MAGs. We randomly selected 18 MAGs in 100 replicates among the remaining MAGs and calculated the number of gene types for each functional category including metal transport and redox, nitrogen and sulfur metabolism, and carbohydrate degradation. The box plots shows data distribution for 100 replicates based on a five number summary. The table on the right compares the number of gene types in each functional profile of dominant MAGs and remaining MAGs.

**Additional file 2:** **Table S1.** Sampling sites and elemental composition of the sediment samples. **Table S2.** Assembly statistics for the seven sediment samples. **Table S3.** All identified SGs and their relative abundance across each sample. **Table S4.** All nonredundant MAGs identified in the study, and associated information. **Table S5.** The relative abundance of MAGs across each sample. **Table S6.** Information of TCDB's annotation in the 179 MAGs. **Table S7.** Information of metal redox annotation in the 179 MAGs. **Table S8.** The list of proteins involved in iron oxidation and iron reduction. **Table S9.** The matrix for the presence or absence of CAZymes in the 179 MAGs. **Table S10.** The proportion of MAGs which contained CAZymes within each phylum. **Table S11.** The matrix for the presence or absence of enzymes involved in the metabolism of small carbon compounds in the 179 MAGs. **Table S12.** The matrix for the presence or absence of nitrogen and sulfur metabolic genes in the 179 MAGs. **Table S13.** Checked annotations involved in metal transport and redox reactions. **Table S14.** Checked annotations involved in nitrogen and sulfur metabolism.

## Data Availability

All clean sequence data, sample information, and MAG sequences from this study have been made publicly available at NCBI, under BioProject ID PRJNA808646. Gene annotation files have been uploaded to the website (https://doi.org/10.5281/zenodo.8015844).
