# Supplementary figures and images for: Microbe-driven elemental cycling enables microbial adaptation to deep-sea ferromanganese nodule sediment fields
Source: Microbiome. 2023 Jul 25;11:160. doi: 10.1186/s40168-023-01601-2 (PMC10367259; doi:10.1186/s40168-023-01601-2)

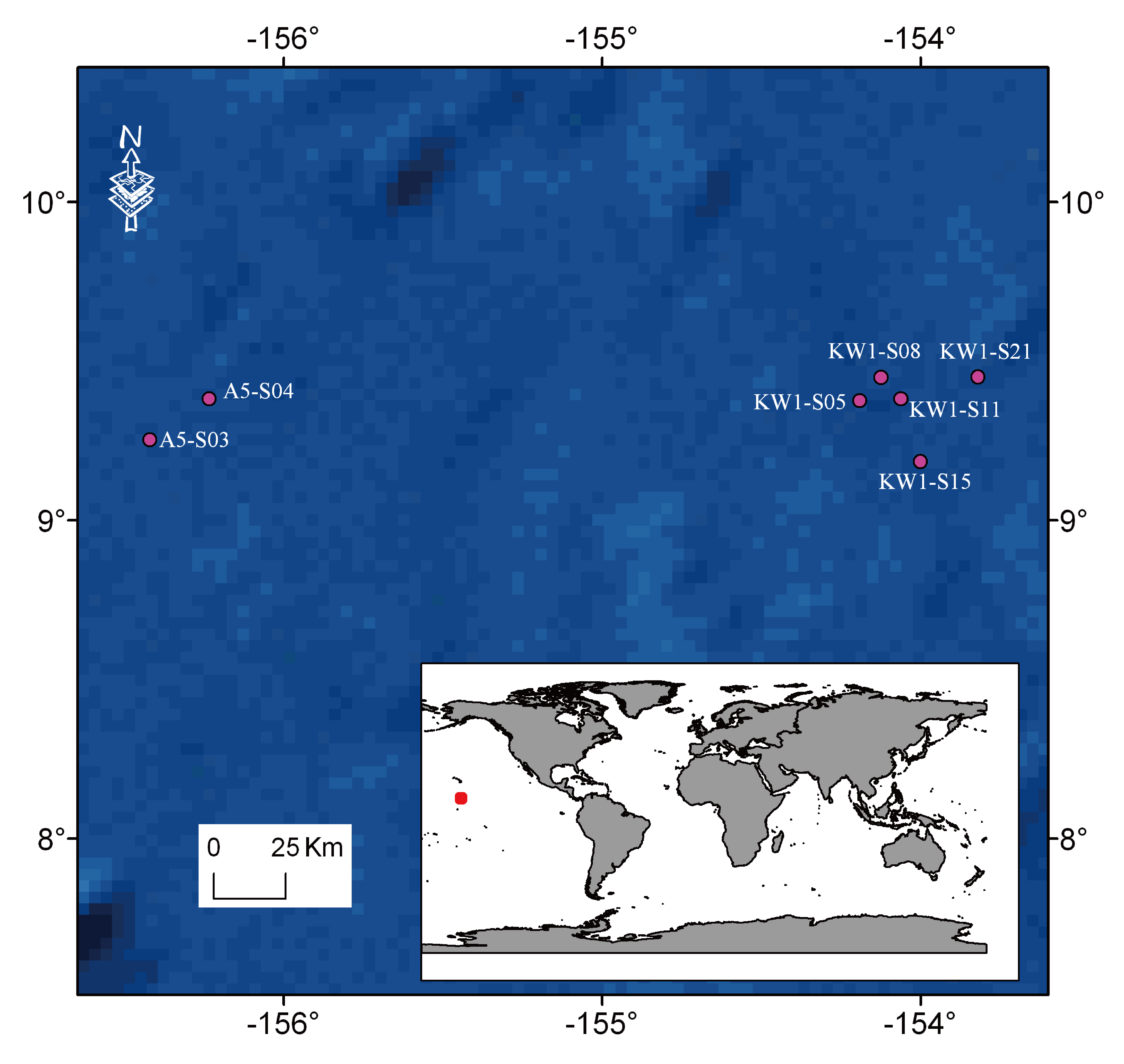

Supplement: Supplementary file 2 — Additional file 1: Fig. S1. Location of sampling sites in the CCFZ polymetallic nodule province in the eastern equatorial Pacific Ocean. Fig. S2. rpS3 species group classification and abundance. a, Relative abundance of all species groups (SGs) in each phylum. Red represent the most abundant SGs, whose combined average relative abundance was at least 25% of that of all SGs. ‘Other’ comprises phyla accounting for ≤ 5 SGs. ‘Unclassify’ indicates that some SGs could not be classified by phylogenetic methods. b, Relative abundance of members of the microbial community at the phylum level across seven samples. Fig. S3. Comparison of GTDB- and rpS3 gene-based classifications of the MAGs. The left side of the Sankey diagram represents the GTDB classification at the phylum level for MAGs containing rpS3 gene sequences. The right side represents rpS3 gene-based classification using NCBI taxonomy. Fig. S4. Schematic diagram of conserved domains of MnxG and MoxA proteins. Blue regions represent cupredoxin superfamily, and purple region represents LysM superfamily. Fig. S5. Flavin-based extracellular electron transfer (EET) in MAGs. a, EET model with iron as an electron acceptor. The process of electron transfer was drawn in accordance with [58]. In brief, the electron transfer path from NAD to Fe(III) includes Ndh2, DMK, EetB, EetA, and FMN groups on PplA, or free flavin shuttles. b, Synthesis of DMK via the proteins DmkB and DmkA. c, Post-translational modification of PplA. FAD is secreted via FmnA and RibU, and FmnB uses FAD to post-translationally modify PplA. The treemaps indicate the composition of MAGs which contained the genes encoding the proteins. DHNA, 1,4-dihydroxy-2-naphthoyl-CoA; DMK, demethylmenaquinone; FAD, flavin adenine dinucleotide; FMN, Flavin mononucleotide; IPP, isopentenyl pyrophosphate. Fig. S6. Functional roles of MAGs in the metabolism of small carbon compounds. The horizontal stacked histograms illustrate the distribution of genomes encoding each fun [file 40168_2023_1601_MOESM1_ESM.zip › Additional file 1 Fig. S1.tif]

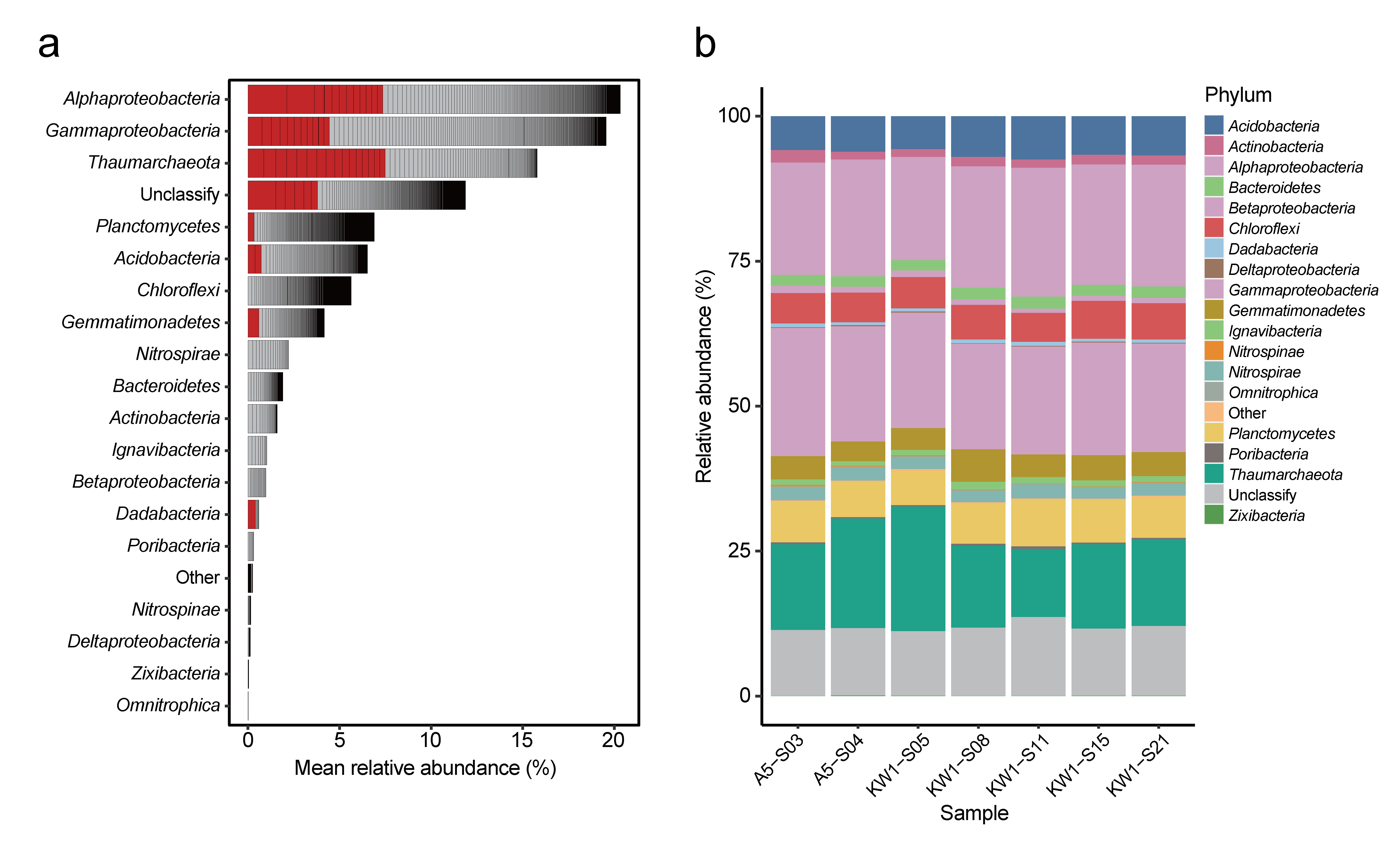

Supplement: Supplementary file 2 — Additional file 1: Fig. S1. Location of sampling sites in the CCFZ polymetallic nodule province in the eastern equatorial Pacific Ocean. Fig. S2. rpS3 species group classification and abundance. a, Relative abundance of all species groups (SGs) in each phylum. Red represent the most abundant SGs, whose combined average relative abundance was at least 25% of that of all SGs. ‘Other’ comprises phyla accounting for ≤ 5 SGs. ‘Unclassify’ indicates that some SGs could not be classified by phylogenetic methods. b, Relative abundance of members of the microbial community at the phylum level across seven samples. Fig. S3. Comparison of GTDB- and rpS3 gene-based classifications of the MAGs. The left side of the Sankey diagram represents the GTDB classification at the phylum level for MAGs containing rpS3 gene sequences. The right side represents rpS3 gene-based classification using NCBI taxonomy. Fig. S4. Schematic diagram of conserved domains of MnxG and MoxA proteins. Blue regions represent cupredoxin superfamily, and purple region represents LysM superfamily. Fig. S5. Flavin-based extracellular electron transfer (EET) in MAGs. a, EET model with iron as an electron acceptor. The process of electron transfer was drawn in accordance with [58]. In brief, the electron transfer path from NAD to Fe(III) includes Ndh2, DMK, EetB, EetA, and FMN groups on PplA, or free flavin shuttles. b, Synthesis of DMK via the proteins DmkB and DmkA. c, Post-translational modification of PplA. FAD is secreted via FmnA and RibU, and FmnB uses FAD to post-translationally modify PplA. The treemaps indicate the composition of MAGs which contained the genes encoding the proteins. DHNA, 1,4-dihydroxy-2-naphthoyl-CoA; DMK, demethylmenaquinone; FAD, flavin adenine dinucleotide; FMN, Flavin mononucleotide; IPP, isopentenyl pyrophosphate. Fig. S6. Functional roles of MAGs in the metabolism of small carbon compounds. The horizontal stacked histograms illustrate the distribution of genomes encoding each fun [file 40168_2023_1601_MOESM1_ESM.zip › Additional file 1 Fig. S2.tif]

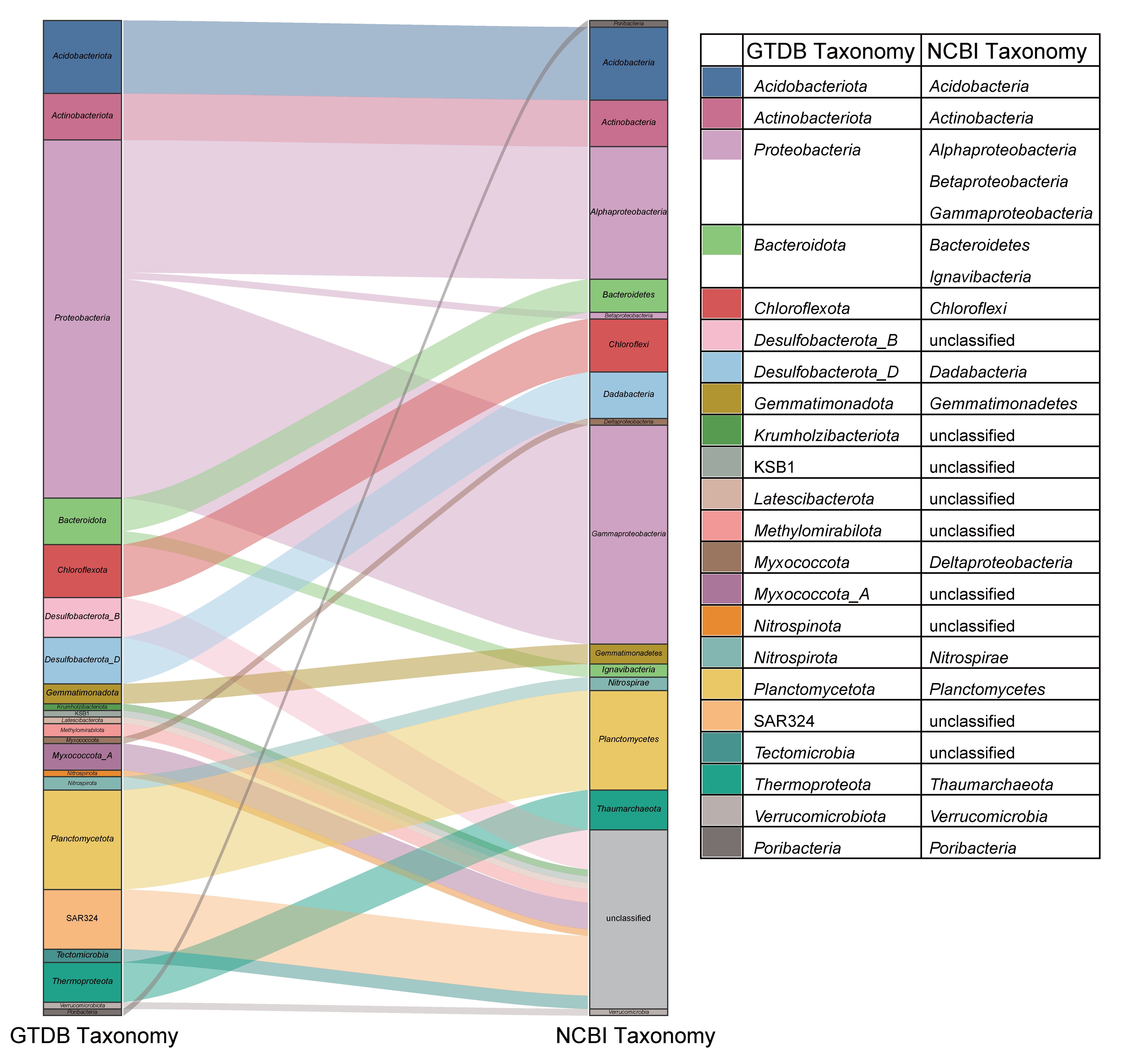

Supplement: Supplementary file 2 — Additional file 1: Fig. S1. Location of sampling sites in the CCFZ polymetallic nodule province in the eastern equatorial Pacific Ocean. Fig. S2. rpS3 species group classification and abundance. a, Relative abundance of all species groups (SGs) in each phylum. Red represent the most abundant SGs, whose combined average relative abundance was at least 25% of that of all SGs. ‘Other’ comprises phyla accounting for ≤ 5 SGs. ‘Unclassify’ indicates that some SGs could not be classified by phylogenetic methods. b, Relative abundance of members of the microbial community at the phylum level across seven samples. Fig. S3. Comparison of GTDB- and rpS3 gene-based classifications of the MAGs. The left side of the Sankey diagram represents the GTDB classification at the phylum level for MAGs containing rpS3 gene sequences. The right side represents rpS3 gene-based classification using NCBI taxonomy. Fig. S4. Schematic diagram of conserved domains of MnxG and MoxA proteins. Blue regions represent cupredoxin superfamily, and purple region represents LysM superfamily. Fig. S5. Flavin-based extracellular electron transfer (EET) in MAGs. a, EET model with iron as an electron acceptor. The process of electron transfer was drawn in accordance with [58]. In brief, the electron transfer path from NAD to Fe(III) includes Ndh2, DMK, EetB, EetA, and FMN groups on PplA, or free flavin shuttles. b, Synthesis of DMK via the proteins DmkB and DmkA. c, Post-translational modification of PplA. FAD is secreted via FmnA and RibU, and FmnB uses FAD to post-translationally modify PplA. The treemaps indicate the composition of MAGs which contained the genes encoding the proteins. DHNA, 1,4-dihydroxy-2-naphthoyl-CoA; DMK, demethylmenaquinone; FAD, flavin adenine dinucleotide; FMN, Flavin mononucleotide; IPP, isopentenyl pyrophosphate. Fig. S6. Functional roles of MAGs in the metabolism of small carbon compounds. The horizontal stacked histograms illustrate the distribution of genomes encoding each fun [file 40168_2023_1601_MOESM1_ESM.zip › Additional file 1 Fig. S3.tif]

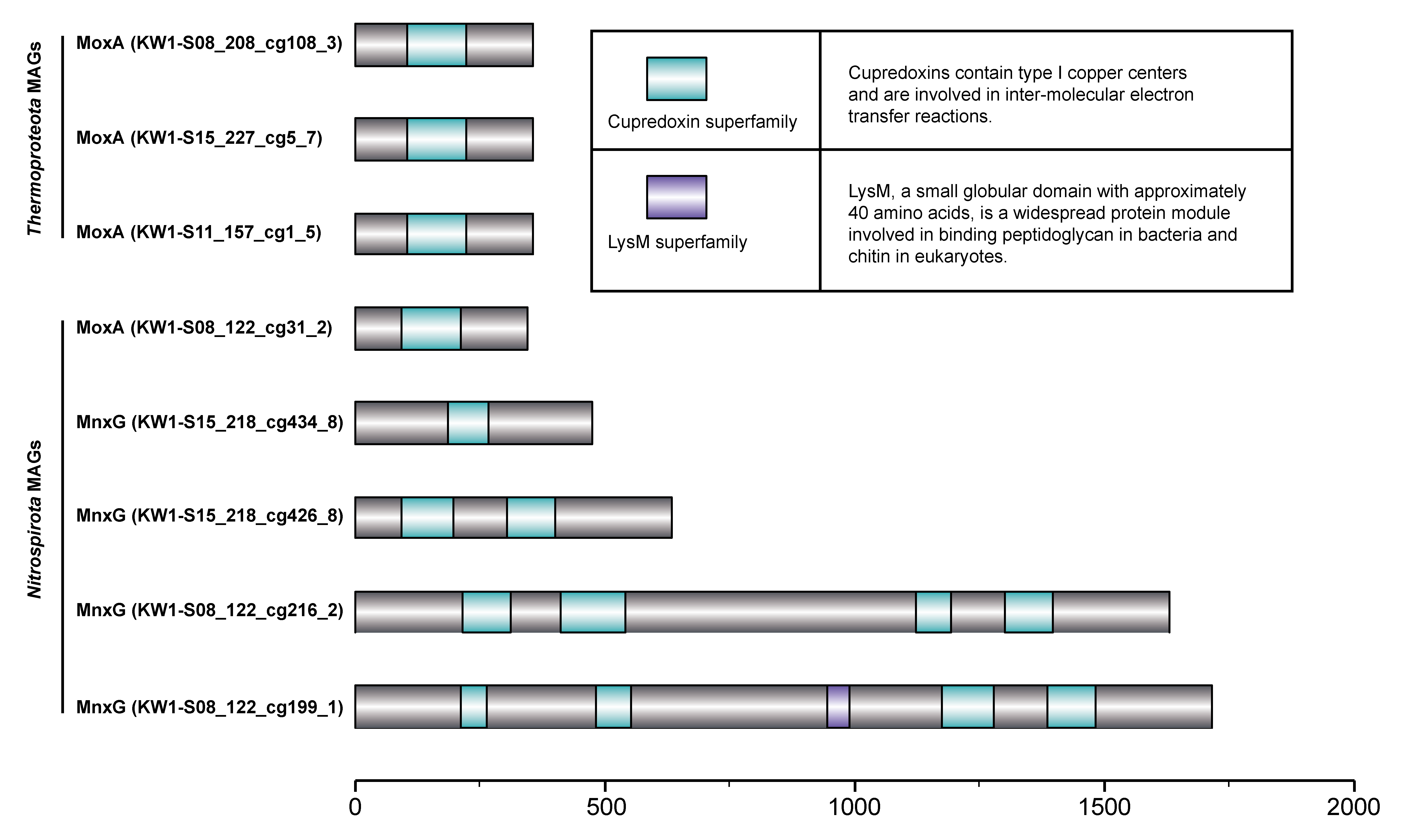

Supplement: Supplementary file 2 — Additional file 1: Fig. S1. Location of sampling sites in the CCFZ polymetallic nodule province in the eastern equatorial Pacific Ocean. Fig. S2. rpS3 species group classification and abundance. a, Relative abundance of all species groups (SGs) in each phylum. Red represent the most abundant SGs, whose combined average relative abundance was at least 25% of that of all SGs. ‘Other’ comprises phyla accounting for ≤ 5 SGs. ‘Unclassify’ indicates that some SGs could not be classified by phylogenetic methods. b, Relative abundance of members of the microbial community at the phylum level across seven samples. Fig. S3. Comparison of GTDB- and rpS3 gene-based classifications of the MAGs. The left side of the Sankey diagram represents the GTDB classification at the phylum level for MAGs containing rpS3 gene sequences. The right side represents rpS3 gene-based classification using NCBI taxonomy. Fig. S4. Schematic diagram of conserved domains of MnxG and MoxA proteins. Blue regions represent cupredoxin superfamily, and purple region represents LysM superfamily. Fig. S5. Flavin-based extracellular electron transfer (EET) in MAGs. a, EET model with iron as an electron acceptor. The process of electron transfer was drawn in accordance with [58]. In brief, the electron transfer path from NAD to Fe(III) includes Ndh2, DMK, EetB, EetA, and FMN groups on PplA, or free flavin shuttles. b, Synthesis of DMK via the proteins DmkB and DmkA. c, Post-translational modification of PplA. FAD is secreted via FmnA and RibU, and FmnB uses FAD to post-translationally modify PplA. The treemaps indicate the composition of MAGs which contained the genes encoding the proteins. DHNA, 1,4-dihydroxy-2-naphthoyl-CoA; DMK, demethylmenaquinone; FAD, flavin adenine dinucleotide; FMN, Flavin mononucleotide; IPP, isopentenyl pyrophosphate. Fig. S6. Functional roles of MAGs in the metabolism of small carbon compounds. The horizontal stacked histograms illustrate the distribution of genomes encoding each fun [file 40168_2023_1601_MOESM1_ESM.zip › Additional file 1 Fig. S4.tif]

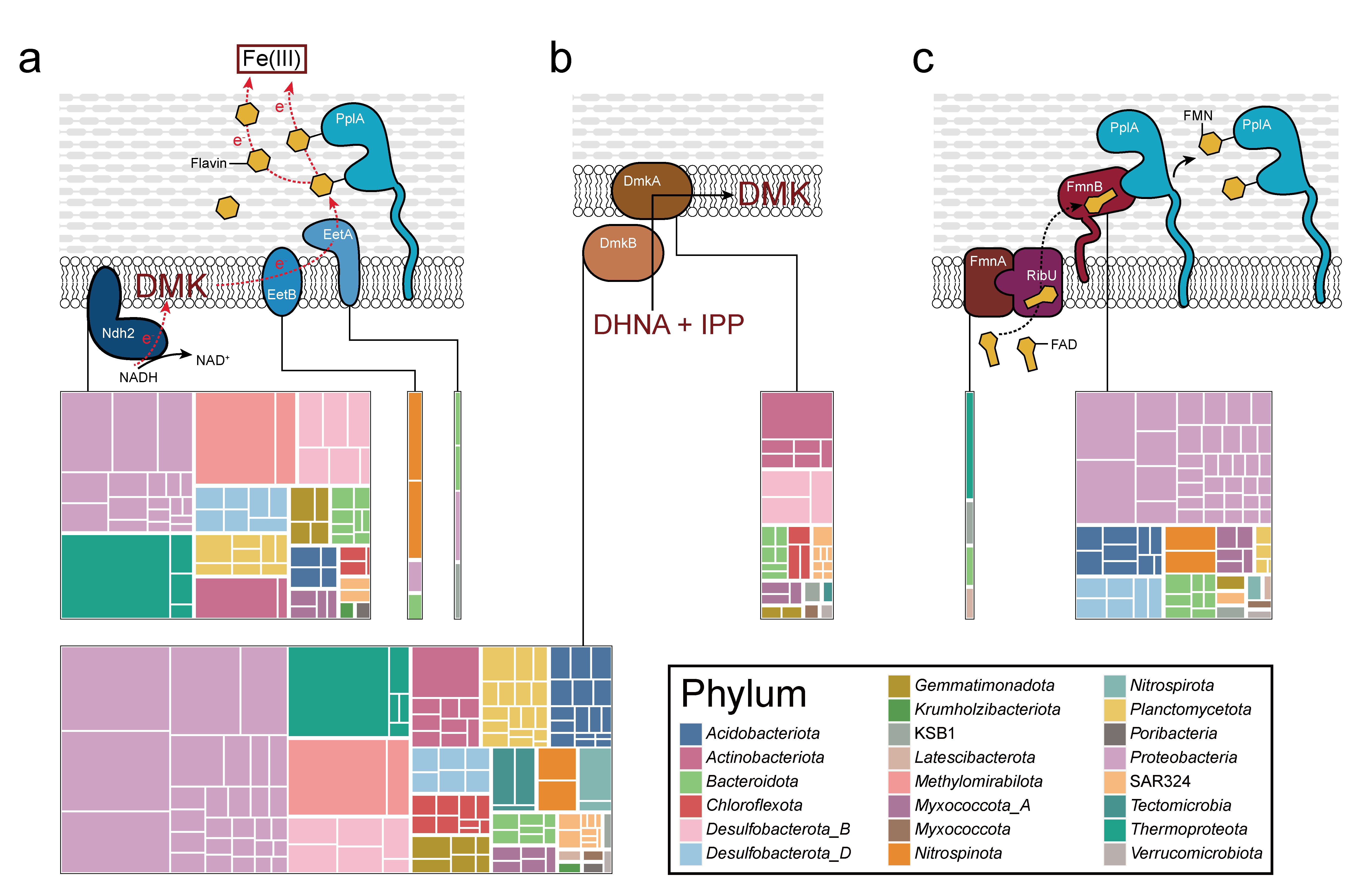

Supplement: Supplementary file 2 — Additional file 1: Fig. S1. Location of sampling sites in the CCFZ polymetallic nodule province in the eastern equatorial Pacific Ocean. Fig. S2. rpS3 species group classification and abundance. a, Relative abundance of all species groups (SGs) in each phylum. Red represent the most abundant SGs, whose combined average relative abundance was at least 25% of that of all SGs. ‘Other’ comprises phyla accounting for ≤ 5 SGs. ‘Unclassify’ indicates that some SGs could not be classified by phylogenetic methods. b, Relative abundance of members of the microbial community at the phylum level across seven samples. Fig. S3. Comparison of GTDB- and rpS3 gene-based classifications of the MAGs. The left side of the Sankey diagram represents the GTDB classification at the phylum level for MAGs containing rpS3 gene sequences. The right side represents rpS3 gene-based classification using NCBI taxonomy. Fig. S4. Schematic diagram of conserved domains of MnxG and MoxA proteins. Blue regions represent cupredoxin superfamily, and purple region represents LysM superfamily. Fig. S5. Flavin-based extracellular electron transfer (EET) in MAGs. a, EET model with iron as an electron acceptor. The process of electron transfer was drawn in accordance with [58]. In brief, the electron transfer path from NAD to Fe(III) includes Ndh2, DMK, EetB, EetA, and FMN groups on PplA, or free flavin shuttles. b, Synthesis of DMK via the proteins DmkB and DmkA. c, Post-translational modification of PplA. FAD is secreted via FmnA and RibU, and FmnB uses FAD to post-translationally modify PplA. The treemaps indicate the composition of MAGs which contained the genes encoding the proteins. DHNA, 1,4-dihydroxy-2-naphthoyl-CoA; DMK, demethylmenaquinone; FAD, flavin adenine dinucleotide; FMN, Flavin mononucleotide; IPP, isopentenyl pyrophosphate. Fig. S6. Functional roles of MAGs in the metabolism of small carbon compounds. The horizontal stacked histograms illustrate the distribution of genomes encoding each fun [file 40168_2023_1601_MOESM1_ESM.zip › Additional file 1 Fig. S5.tif]

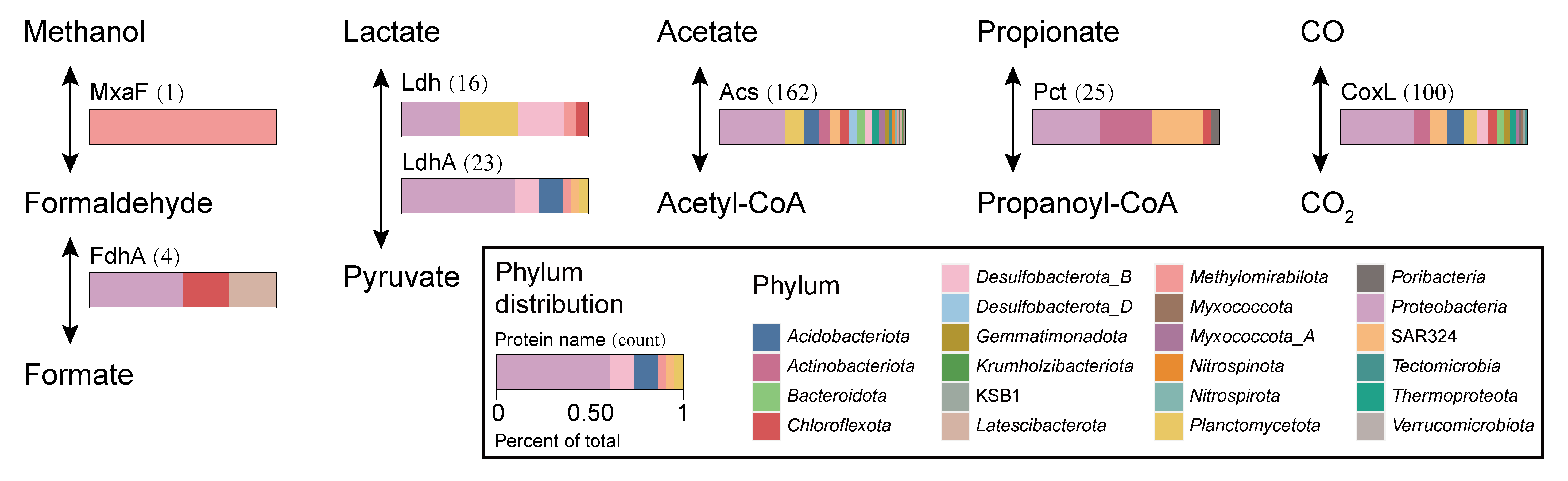

Supplement: Supplementary file 2 — Additional file 1: Fig. S1. Location of sampling sites in the CCFZ polymetallic nodule province in the eastern equatorial Pacific Ocean. Fig. S2. rpS3 species group classification and abundance. a, Relative abundance of all species groups (SGs) in each phylum. Red represent the most abundant SGs, whose combined average relative abundance was at least 25% of that of all SGs. ‘Other’ comprises phyla accounting for ≤ 5 SGs. ‘Unclassify’ indicates that some SGs could not be classified by phylogenetic methods. b, Relative abundance of members of the microbial community at the phylum level across seven samples. Fig. S3. Comparison of GTDB- and rpS3 gene-based classifications of the MAGs. The left side of the Sankey diagram represents the GTDB classification at the phylum level for MAGs containing rpS3 gene sequences. The right side represents rpS3 gene-based classification using NCBI taxonomy. Fig. S4. Schematic diagram of conserved domains of MnxG and MoxA proteins. Blue regions represent cupredoxin superfamily, and purple region represents LysM superfamily. Fig. S5. Flavin-based extracellular electron transfer (EET) in MAGs. a, EET model with iron as an electron acceptor. The process of electron transfer was drawn in accordance with [58]. In brief, the electron transfer path from NAD to Fe(III) includes Ndh2, DMK, EetB, EetA, and FMN groups on PplA, or free flavin shuttles. b, Synthesis of DMK via the proteins DmkB and DmkA. c, Post-translational modification of PplA. FAD is secreted via FmnA and RibU, and FmnB uses FAD to post-translationally modify PplA. The treemaps indicate the composition of MAGs which contained the genes encoding the proteins. DHNA, 1,4-dihydroxy-2-naphthoyl-CoA; DMK, demethylmenaquinone; FAD, flavin adenine dinucleotide; FMN, Flavin mononucleotide; IPP, isopentenyl pyrophosphate. Fig. S6. Functional roles of MAGs in the metabolism of small carbon compounds. The horizontal stacked histograms illustrate the distribution of genomes encoding each fun [file 40168_2023_1601_MOESM1_ESM.zip › Additional file 1 Fig. S6.tif]

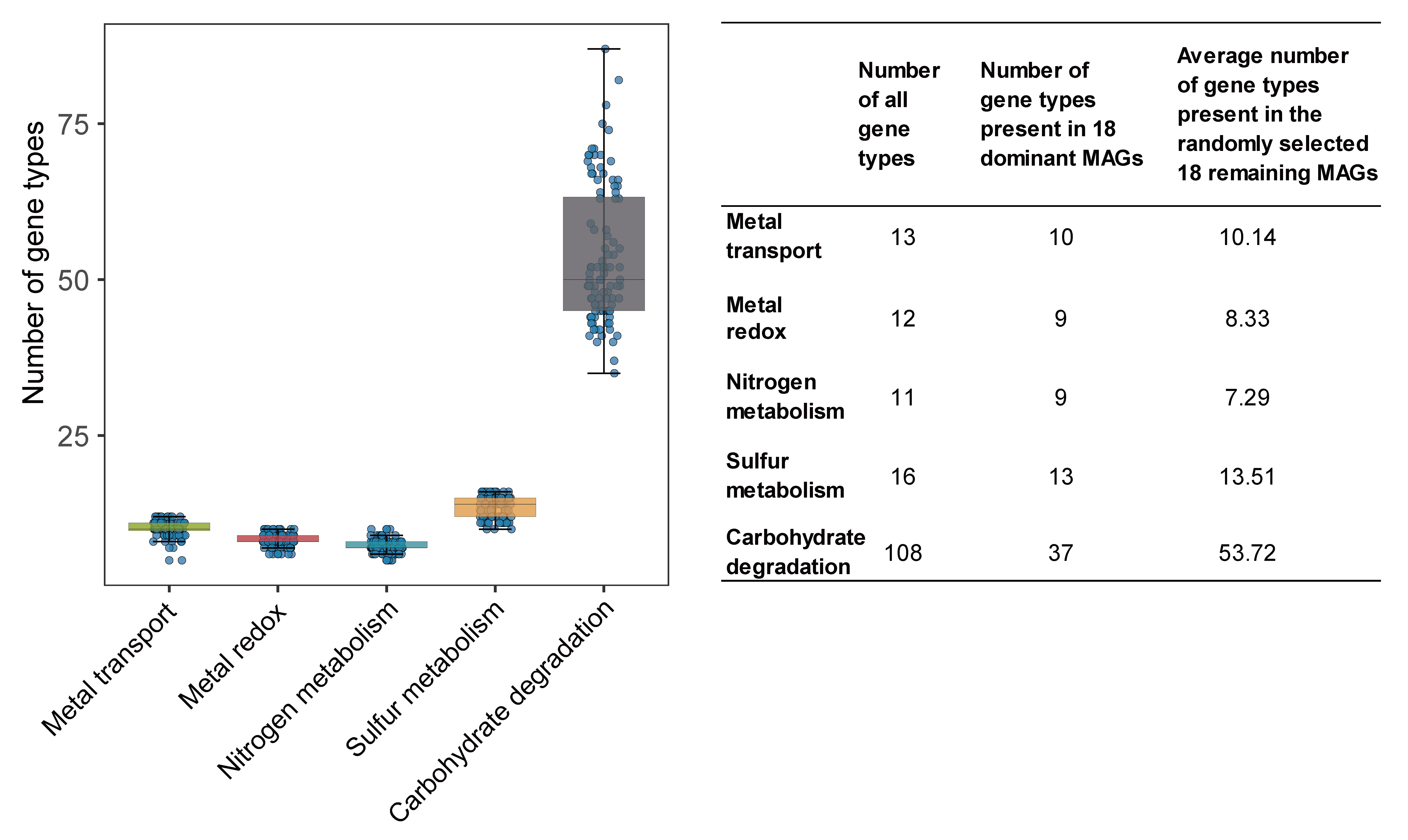

Supplement: Supplementary file 2 — Additional file 1: Fig. S1. Location of sampling sites in the CCFZ polymetallic nodule province in the eastern equatorial Pacific Ocean. Fig. S2. rpS3 species group classification and abundance. a, Relative abundance of all species groups (SGs) in each phylum. Red represent the most abundant SGs, whose combined average relative abundance was at least 25% of that of all SGs. ‘Other’ comprises phyla accounting for ≤ 5 SGs. ‘Unclassify’ indicates that some SGs could not be classified by phylogenetic methods. b, Relative abundance of members of the microbial community at the phylum level across seven samples. Fig. S3. Comparison of GTDB- and rpS3 gene-based classifications of the MAGs. The left side of the Sankey diagram represents the GTDB classification at the phylum level for MAGs containing rpS3 gene sequences. The right side represents rpS3 gene-based classification using NCBI taxonomy. Fig. S4. Schematic diagram of conserved domains of MnxG and MoxA proteins. Blue regions represent cupredoxin superfamily, and purple region represents LysM superfamily. Fig. S5. Flavin-based extracellular electron transfer (EET) in MAGs. a, EET model with iron as an electron acceptor. The process of electron transfer was drawn in accordance with [58]. In brief, the electron transfer path from NAD to Fe(III) includes Ndh2, DMK, EetB, EetA, and FMN groups on PplA, or free flavin shuttles. b, Synthesis of DMK via the proteins DmkB and DmkA. c, Post-translational modification of PplA. FAD is secreted via FmnA and RibU, and FmnB uses FAD to post-translationally modify PplA. The treemaps indicate the composition of MAGs which contained the genes encoding the proteins. DHNA, 1,4-dihydroxy-2-naphthoyl-CoA; DMK, demethylmenaquinone; FAD, flavin adenine dinucleotide; FMN, Flavin mononucleotide; IPP, isopentenyl pyrophosphate. Fig. S6. Functional roles of MAGs in the metabolism of small carbon compounds. The horizontal stacked histograms illustrate the distribution of genomes encoding each fun [file 40168_2023_1601_MOESM1_ESM.zip › Additional file 1 Fig. S7.tif]
